# Exhaustive sampling of the fragment space associated to a molecule leading to the generation of conserved fragments

**DOI:** 10.1111/cbdd.13129

**Published:** 2017-12-12

**Authors:** Kathrin Heikamp, Fabio Zuccotto, Michael Kiczun, Peter Ray, Ian H. Gilbert

**Affiliations:** ^1^ Drug Discovery Unit Division of Biological Chemistry and Drug Discovery School of Life Sciences University of Dundee Dundee Scotland, UK

**Keywords:** algorithm development, chemical space analysis, fragment based drug discovery, molecular fragmentation

## Abstract

The first step in hit optimization is the identification of the pharmacophore, which is normally achieved by deconstruction of the hit molecule to generate “deletion analogues.” In silico fragmentation approaches often focus on the generation of small fragments that do not describe properly the fragment space associated to the deletion analogues. We present significant modifications to the molecular fragmentation programme molblocks, which allows the exhaustive sampling of the fragment space associated with a molecule to generate all possible molecular fragments. This generates larger fragments, by combining the smallest fragments. Additionally, it has been modified to deal with the problem of changing pharmacophoric properties through fragmentation, by highlighting bond cuts. The modified molblocks programme was used on a set of drug compounds, where it generated more unique fragments than standard fragmentation approaches by increasing the number of fragments derived per compound. This fragment set was found to be more diverse than those generated by standard fragmentation programmes and was relevant to drug discovery as it contains the key fragments representing the pharmacophoric elements associated with ligand recognition. The use of dummy atoms to highlight bond cuts further increases the information content of fragments by visualizing their previous bonding pattern.

## 1 INTRODUCTION

Fragment‐based drug discovery (FBDD) has become an increasingly common and powerful tool in the drug discovery process.^**[**^ 1, 2, 3 ^**]**^ FBDD is driven by two concepts that explain its use and the reason for its success. Chemical space can be probed more efficiently using a library of small fragments rather than larger molecules. With decreasing size of the molecules, the number of possible molecules in the chemical space decreases as well in an exponential manner. Therefore, a collection of low molecular weight molecules can cover more diversity of chemical space than a more conventional lead‐ or drug‐like library, containing a similar number of compounds.^4^, ^5^, ^6^, ^7^ Moreover, the hit rate for screening fragments should be higher as a small molecule has a higher probability of binding in a receptor without unfavorable interactions.^1^, ^6^ Although the overall potency is typically smaller than for larger molecules, the ligand efficiency (binding efficiency per heavy atom) can be much higher.^5^, ^7^, ^8^ Once suitable fragments are identified, they can be optimized by merging, linking or growing to generate drug leads.^1^, ^7^, ^9^ Care has to be taken to keep the optimum interaction between the core‐fragment and protein as the fragment is grown to make a more potent compound. FBDD has been applied to a wide range of targets and in the context of different diseases.^10^ One of the first success stories was the approval of Vemurafenib, a B‐Raf kinase inhibitor, considered to be the first molecule developed from a FBDD programme to reach FDA approval and the market.^11^ However, FBDD is still not an integral part of drug discovery efforts to target all disease areas; one of the key requirements is to be able to rapidly generate cocrystal structures of the protein and the fragments as they are optimized.^12^

The first step of FBDD is the construction of a fragment library. One approach for fragment library design is to make use of known chemical and biological data and to deconstruct ligands into those fragments that most likely are responsible for the bioactivity, that is the key pharmacophores of the ligands.^2^ For example, a fragment library derived from known ligands was used to generate two receptor‐positive allosteric modulators with enhanced potency for preventing neuroapoptosis.^13^ A library for screening should be diverse and of sufficient size to cover as much chemical space as possible. Libraries are selected based on the physicochemical properties of fragments; typically, the rule‐of‐three^14^ is used, which takes account of molecular weight, lipophilicity (clogP), numbers of hydrogen bond donors and acceptors. High aqueous solubility is also critical for a fragment library.^2^, ^5^ Reactive and toxic functional groups should also be avoided. To determine fragment properties and coverage of chemical space, fragment libraries are typically generated virtually at first.

One method to generate fragment libraries is by fragmentation of a library of larger molecules (e.g., drugs or natural products) that have biological activities. There are a few programmes available that apply different fragmentation rules to deconstruct ligands into smaller molecules. One of the most popular fragmentation methods is the retrosynthetic combinatorial analysis procedure (RECAP) developed in GSK which generates fragments by breaking bonds formed by chemical reactions such as amide, amine and ester.^15^ Several implementations of RECAP can be found in commercial programmes: the fragmenter provided by chemaxon (www.chemaxon.com); the fragment searching and analogue design tool brood distributed by openeye (www.eyesopen.com/brood); and the open‐source RDKit library (www.rdkit.org). The latter also includes an implementation of the “breaking of retrosynthetically interesting chemical substructures” (BRICS) fragmentation that covers a more elaborated set of fragmentation rules along synthetically accessible bonds and generates more fragments than RECAP.^16^

Another approach for fragment generation is to use PUG SOAP, a web tool for the pubchem database^17^ and its functionality. Ligand substructures can be derived using the pubchem superstructure search function in PUG SOAP. The derived fragments are substructures of molecules registered in pubchem; therefore, the fragment space generated is strongly biased to what is in pubchem at any time. In a recent publication, Firth et al.^18^ use SynDir, a fragmentation programme applying ordered set of rules, to generate a fragment library of synthetic fragments.

In addition to these approaches, Ghersi and Singh recently published a suite of programmes called molblocks for breaking down molecules and analysis of the resulting fragments.^19^ The open‐source programme fragments molecules according to a set of fragmentation rules that are defined using the SMARTS notation. The fragmentation rules RECAP, BRICS and CCQ (cleaving a bond between two carbon atoms of which at least one is connected to a heteroatom) are already implemented, and an additional feature allows the user to easily expand these rules and define new ones applicable to different compound spaces. Then, a second program called “analyze” can be applied to cluster the resulting fragments and detect statistically enriched fragments.^19^ These functionalities and the ability to customize the algorithm make molblocks an attractive tool to analyze the fragment spaces derived by ligands having different bioactivities.

The computational approaches described above have been designed to fragment molecules according to synthetic accessibility. Another important consideration when fragmenting molecules is capturing the pharmacophore(s) present in the parent molecules. The pharmacophoric elements of a compound are the chemical features of the molecule responsible for the recognition event of the molecule by its biological target. Yet, one disadvantage of most fragmentation approaches is that the fragmentation can break bonds that might be part of a pharmacophore altering its very nature. For example, an amide group, acting as both an H‐bond donor and H‐bond acceptor, defines a very specific pharmacophore. However, the amide group is normally cleaved during fragmentation as synthetically it will be made by coupling a carboxylic acid with an amine, neither of which retains the pharmacophoric pattern of the amide.

In this paper, we present a modified version of the molblocks programme suite^19^ developed to exhaustively generate all possible fragments related to a given compound and to account for the limitations of current fragmentation programs. The fragmentation method currently implemented in molblocks will result in a set of building blocks that can be used to synthetically access the parent compound by following a set of conventional chemical reactions coded in the fragmentation rules. As we are interested in capturing the pharmacophore content of a given molecule, we worked on the development of a tool to generate smaller fragments containing different combinations of the pharmacophoric elements present on the parent compound. Therefore, the code of the “fragment” programme of molblocks was modified to allow an exhaustive sampling of the fragment space associated to a compound. Additionally, dummy atoms were introduced to mark points where the compounds were broken down to highlight bond cuts and maintain information about the molecular pharmacophore. The modifications were restricted to the fragmentation part of the programme suite and are discussed in the Section 2 part. The analysis functionality of molblocks was retained and can be applied as presented by Ghersi and Singh.^19^ The modified programme was used to fragment known drugs to determine the range of fragments that could be derived from known drugs. The programme could also have utility in designing or enhancing a fragment library and in the analysis of hits from a screen.

## 2 METHODS AND MATERIALS

### 2.1 Increasing fragment space

To increase the fragment space created, the molblocks suite was modified using two different approaches. First, it was amended to create larger and more complex fragments. In addition, the fragmentation rules were extended allowing more bonds to be cut. The former is described in this section.

The main modification of the molblocks programme was the introduction of an exhaustive fragmentation to derive, for a given ligand, combinatorically all possible molecular fragments. Like other fragmentation methods, molblocks generates the smallest fragments that are derived by applying a set of fragmentation rules. However, in some cases, it might be useful to create not just the smallest possible fragments but some intermediate fragments as well, for example in cases where a key pharmacophore is known to contain more than one functional group. Pharmacophores could still be obtained by modifying the rules for each compound individually, but require the application on a compound level to make sure that all key fragments are derived. The fragmentation of large compound libraries will then require more time.

**Figure 1:**
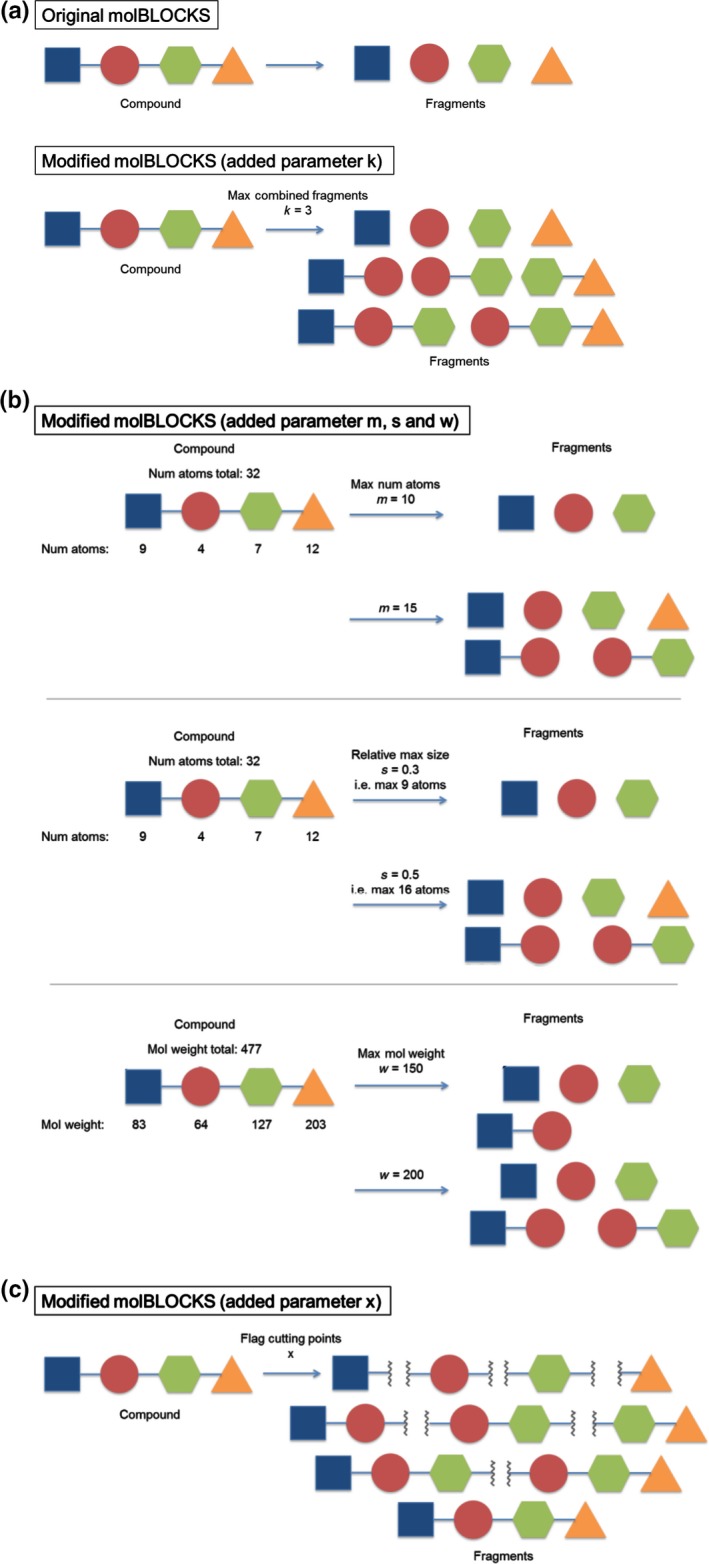
Modifications to the molblocks suite. Schematic explanation of the parameters introduced to modify the original molblocks program. The figure explains the five parameters added as follows: (a) *k* for exhaustive fragmentation, (b) fragment filtering parameters for maximal size (*m*), relative size to parent ligand (*s*), and molecular weight (*w*), (c) *x* flag for highlighting of cutting points [Colour figure can be viewed at wileyonlinelibrary.com]

The exhaustive fragmentation is performed in two steps. First, all smallest fragments are generated as implemented in the original version using extensive fragmentation (−*e* flag). Then, these fragments are combined to larger ones through combinatorics, that is each smallest fragment is combined with its neighbouring fragment to generate larger subgroups. The new parameter “*k*” specifies how many of the smallest fragments should be maximally combined. If the parameter is set to 1, the output will be the same as using extensive fragmentation in the original implementation. A larger value results in the calculation of all fragments up to the specified number of combined smallest fragment. By this, also “intermediate” fragments are generated. If the parameter is set to a very large (or negative) number, exhaustive fragmentation is performed so that all possible fragments for the specified fragmentation rules are derived. Figure 1a highlights the modifications to the molblocks suite.

Due to these modifications, molecules can be generated that are equal to the input compound (or close in size) and might not necessarily fulfil the standard definition of a fragment. Therefore, three additional parameters are introduced to filter larger fragments. First, fragments can be filtered by specifying the maximal number of atoms a fragment is allowed to have (parameter “*m*”). A second filter (parameter “*s*”) allows the specification of the maximal size relative to the parent compound, for example a fragment is allowed to contain up to 60% of the atoms in the parent molecule, if the value is set to 0.6. A value slightly lower than 1 makes sure that the input compound is not returned as a fragment, and all possible substructures are still retrieved. Finally, the derived fragments can be filtered by molecular weight (parameter “*w*”). This allows, for example the removal of fragments not satisfying the “rule‐of‐three”^14^ molecular weight definition if that is what is required. Figure 1b reports the three different fragment filtering methods.

### 2.2 Preserving pharmacophoric characteristic

Additionally, the programme was modified to address the limitation that the fragment pharmacophore could potentially change compared to the parent ligand. In the original molblocks version, hydrogens are added to those atoms defining the bond that was broken to create the fragments. This approach can increase the number of hydrogen bond donors present, for example if the fragmentation rule was applied to a bond with a nitrogen and another heavy atom. This fragmentation approach may not reproduce the pharmacophore and binding interactions of compounds. To account for this, the programme was extended to allow highlighting of cutting points. A flag named “*x*” can be turned on to replace the added hydrogens with dummy atoms (asterisks) in the smiles representation. The asterisks in the smiles strings represent a new, undefined atom having no specified atom type. By this approach, no assumptions are made about the identity of the atoms on either side of the bonds which have been fragmented. This is a desired functionality as it keeps the programme straightforward, flexible and usable with any fragmentation rule provided as a smarts string. Additionally, the returned fragments have valid smiles strings that can be used in other programmes. Figure 1c shows how fragments with cutting points appear schematically.

### 2.3 Expanding fragmentation rules

Generating the complete molecular fragment space associated with a given molecule cannot only be addressed by increasing the number of fragments through exhaustive fragmentation; the chemical context needs to be considered as well. It is necessary to use fragmentation rules that can deal with the complexity and immense diversity of the chemical space of a compound. Therefore, an extended version of the RECAP rules was created (called extendedRECAP) with an additional 16 rules (see below for download instructions), mostly covering bonds with sulphur, phosphate and other carbon atom environments. These rules were derived through fragmentation of a series of exemplary compounds in house. Although BRICS creates more fragments than the standard RECAP approach, the latter was used as basis to preserve ring structures in the fragments.

The modified molblocks programme and the additional fragmentation rules extendedRECAP are made freely available under https://github.com/kheikamp/modified_molBLOCKS. A description of how to use the programme is included. The programme is available as GPL C++ source code. It can be incorporated into in‐house software, a key advantage over other commercial and proprietary software systems.

### 2.4 Study goal and design

The major focal point of this study was to analyze the influence of the exhaustive fragmentation of compounds on the generated fragment space. The modified molblocks programme will generate many more subgroups of input compounds and potentially a different representation of these subgroups when highlighting the cutting points. This study aimed to identify the changes in the fragments itself and the number of fragments generated per compound and in total. Furthermore, the analysis examined whether key pharmacophores of ligands could be generated using the extended program. For this, the three different fragmentation rules available in molblocks (RECAP, BRICS, and CCQ), and extendedRECAP rules were used. In the analysis, only fragments containing at least five atoms were used (setting “−*n* 5”), and those that are true substructures of the input compounds were generated. The latter was achieved by constraining the fragment size relative to the parent structure to 99% (setting “−*s* 0.99”), that is the fragment was only allowed to contain 99% of the atoms of the parent structure. The number of combined smallest fragments specified through the parameter *k* was either set to 1 (setting “−*k* 1”) to reflect standard fragmentation or to 8 (setting “−*k* 8”) to derive all important subgroups. The empirical value 8 was identified to be appropriate in preliminary in‐house calculations. Finally, the fragmentation was performed both using the dummy atoms flag (“−*x*”) and the default of attaching hydrogens to cutting points. In the following, the settings of “−*n* 5” and “−*s* 0.99” were kept constant for all fragmentation calculations, and only the parameters *k* and *x* and the fragmentation methods were modified.

### 2.5 Compound data sets

For our analysis, drug data were selected from the drugbank 20 and the chembl 21, 22 databases and were processed using knime.^23^ From drugbank, 1,556 compounds were derived (accessed on July 14, 2015). Three drugs were excluded from the analysis as they contained repetitive units with a multiple fragment count and hence did not contain a smiles representation (compound identifiers DB00707, DB00895, and DB06439). The remaining compounds were processed to remove salts using the default settings in the RDKit Salt Stripper node in knime and two additional filters for Gadolinium (Gd+3) and Silver (Ag+) were applied. Canonical smiles were calculated using Open Babel resulting in a set of 1,550 drugs. There were 27 drugs that consisted of more than one fragment, of which only three were kept, as the fragments were the same. To remove the multiple representations, these structures were reduced to its common entity. The final set of compounds from drugbank consisted of 1,526 approved drugs.

From chembl, 1,556 approved drugs with structure information were obtained (Approved Drug Data Freeze: November 2014, accessed on July 14, 2015). The same filtering steps were applied to these drugs resulting in 1,537 drugs without salts (filter removed no drugs) and consisting only of a single structure (filter removed 19 drugs).

A unique set of drug data was derived by merging drugbank and chembl filtered drugs on their canonical smiles calculated with Open Babel. The total number of 2,047 unique drugs obtained was further processed by an inorganic filter and compounds with a molecular weight larger than 600 Da were removed. The final set consisted of 1,762 drugs in total having different areas of application.

## 3 RESULTS AND DISCUSSION

### 3.1 Intermediate fragments generation

The fragmentation using the four different fragmentation rules BRICS, CCQ, RECAP and extendedRECAP, and the parameters specified in the Methods and Materials section was applied to the combined set of drugs from drugbank and chembl to analyze the effect of exhaustive fragmentation. Some of the compounds were not fragmented, either because the fragmentation rules did not match any part of structure or the generated fragments were too small and did not fulfil the condition of having at least five atoms. Table 1 lists the number of drugs that could be fragmented using the specified parameters and the number of fragments that were generated from this set. It can be seen that more compounds were fragmented when the combinatorial factor was set to 8. This means that some of the compounds generated too small fragments that were filtered out when using the simple fragmentation. The combinatorial fragmentation with the modified molblocks programme, however, produced intermediate fragments that were large enough to pass the filter of minimal number of atoms in the fragments. Furthermore, the number of unique fragments generated by this is much larger than using the simple fragmentation. The same trends can be seen for the four different fragmentation rules and differs in the multiplier. The multiplier is between 20 when using RECAP and 120 when using the extendedRECAP rules and dummy atoms.

**Table 1:** Number of fragmented drugs and generated fragments

Parameter	No. of fragmented drugs	No. of unique frags
BRICS, *n* = 5, *k* = 1, *s* = 0.99	1,564	961
BRICS, *n* = 5, *k* = 8, *s* = 0.99	1,599	56,008
BRICS, *n* = 5, *k* = 8, *s* = 0.99, *x*	1,599	67,546
CCQ, *n* = 5, *k* = 1, *s* = 0.99	1,462	1,121
CCQ, *n* = 5, *k* = 8, *s* = 0.99	1,543	59,081
CCQ, *n* = 5, *k* = 8, *s* = 0.99, *x*	1,543	69,094
RECAP, *n* = 5, *k* = 1, *s* = 0.99	1,561	1,395
RECAP, *n* = 5, *k* = 8, *s* = 0.99	1,587	28,082
RECAP, *n* = 5, *k* = 8, *s* = 0.99, *x*	1,587	29,992
extendedRECAP, *n* = 5, *k* = 1, *s* = 0.99	1,676	620
extendedRECAP, *n* = 5, *k* = 8, *s* = 0.99	1,709	54,888
extendedRECAP, *n* = 5, *k* = 8, *s* = 0.99, *x*	1,709	74,001

The table lists the number of drugbank and chembl drugs that were fragmented and the number of generated fragments for the four applied fragmentation rules BRICS, CCQ, RECAP and extendedRECAP. The parameter settings of (*n* = 5) for minimal number of atoms and (*s* = 0.99) for the relative maximal size were kept constant for all fragmentation calculations. For each rule, three different parameter settings were chosen to represent simple fragmentation (*k* = 1) and exhaustive fragmentation with (*k* = 8, *x*) and without dummy atoms (*k* = 8).

**Figure 2:**
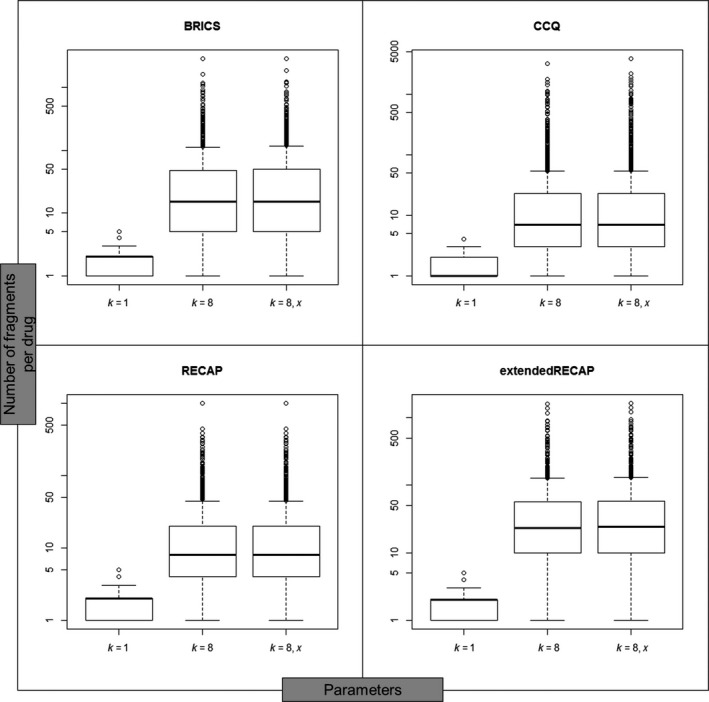
Distribution of fragments per drug. Distribution of the number of fragments per drug compound for the four different fragmentation rules BRICS, CCQ, RECAP and extendedRECAP. For each rule, three different parameter settings were chosen to represent simple fragmentation (*k* = 1) and exhaustive fragmentation with (*k* = 8, *x*) and without dummy atoms (*k* = 8)

The higher number of unique fragments from the drug set generated by “extendedRECAP” is a result of an increasing number of fragments generated for each drug compound. There are more fragmented drugs as more fragments fulfil the required minimal number of five atoms. Figure 2 shows the number of fragments per drug as boxplots for the different fragmentation rules and parameters. The figure highlights that the distribution of the number of fragments and median values are comparable for all four fragmentation rules. Increasing the combinatorial parameter *k* from simple to exhaustive fragmentation increases the quartiles and median number of fragments per drug compound. Thereby, the introduction of dummy atoms has only a small influence on the fragment frequency. The lowest increments of median values can be seen for the fragmentation methods CCQ and RECAP with an increase from one to seven and two to eight when applying a combinatorial factor *k* = 8, respectively. Although CCQ has the lowest median values, it also results in most of the outliers and highest number of fragments. The maximum number of fragments per compound is 3819 when using a value *k* = 8 and dummy atoms together with the rule CCQ. This is why the second highest number of unique fragments is obtained using this method. More unique fragments are obtained with the extendedRECAP method. Here, the median values of 23 (*k* = 8) and 24 (*k* = 8, *x*) are higher than for all other fragmentation methods.

**Figure 3:**
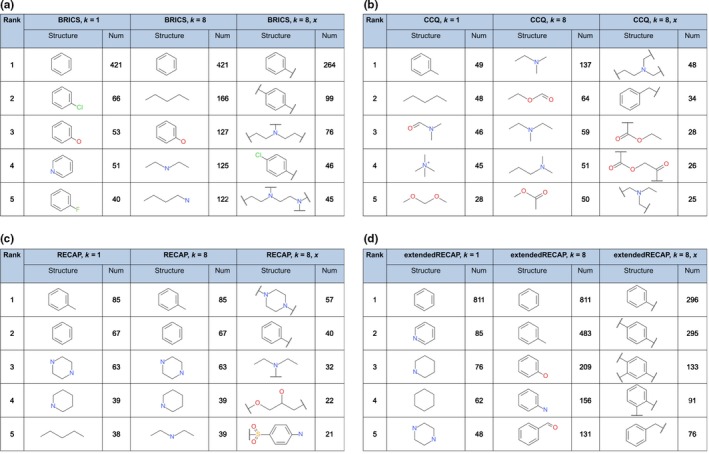
Top‐ranked fragments. The structures of the five most frequent fragments and the number of occurrences (Num) are reported. Data are shown for simple fragmentation (*k* = 1) and exhaustive fragmentation with (*k* = 8, *x*) and without dummy atoms (*k* = 8) for the four fragmentation methods BRICS (a), CCQ (b), RECAP (c) and extendedRECAP (d) [Colour figure can be viewed at wileyonlinelibrary.com]

The exhaustive fragmentation also has an influence on the top‐ranked fragments per fragmentation method. Figure 3 shows the five most frequent fragments for each of the four fragmentation methods and for simple and exhaustive fragmentation. The smiles of the 100 most frequent reported in the Tables S1–S12 of the Supporting information.

The figures highlight that both the introduction of the combinatorial factor and the dummy atoms have an influence on the most frequent fragments. In Figure 3a, the parameter *k* = 8 increases the number of aliphatic atom chains for BRICS that are a result of combining individual atoms and small fragments. Highlighting the cutting points by adding the flag *x* returns a better illustration of the original molecular structures that were fragmented. For example, the top‐ranked benzene ring is now separated into two structures with a different number of cutting points. In general, slight changes in the order can be seen for changing the parameters. The most frequent fragments obtained by the fragmentation rule CCQ (Figure 3b) are different to BRICS. Apart from two toluene fragments, all structures are aliphatic chains mostly containing nitrogen or oxygen. The different parameter settings have a strong impact on the top‐ranked fragments and change their order totally. Not shown here is that many additional fragments created with BRICS are due to the separation of ring structures and their recombinations. They are ranked lower as they are less common in the data set. Figure 3c shows the most frequent fragments for the RECAP method. Increasing the combinatorial factor *k* has almost no influence on the five most frequent fragments. The introduction of cutting points reduces the frequency of each fragment and results in complex structures and more carbon chains. Finally, Figure 3d reports the top five fragments for the extendedRECAP fragmentation method. The exhaustive fragmentation results in more aromatic ring structures that are ranked highest. As a result, the two approaches simple and exhaustive fragmentation differs from rank two on. Using exhaustive fragmentation and dummy atoms has a strong impact on the fragment frequencies compared to simple fragmentation. The top four fragments are all benzene structures with a different number and distribution of cutting points. The fifth most frequent structure is toluene. To analyze the impact of the introduced dummy atoms, the fragment cutting points are discussed in the following section 3.3.

### 3.2 Highlighting of cutting points

**Figure 4:**
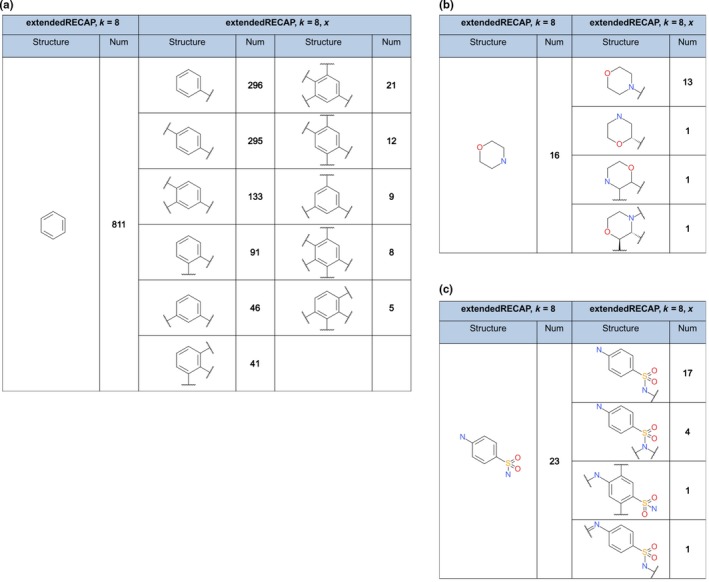
Comparison of fragments with and without cutting points. Fragments generated with dummy atoms are contrasted with fragments generated without cutting points. All fragments are generated with the extendedRECAP and exhaustive fragmentation. The comparison is reported for the three examples: benzene (a), morpholine (b) and sulphonamide (c) [Colour figure can be viewed at wileyonlinelibrary.com]

The previous analysis has already shown that the use of dummy atoms results in more generated fragments. The question is whether these fragments contain more information about the structure they originate from. Figure 4 shows three examples of fragments generated with the extendedRECAP method and a combinatorial factor of 8. In one case, dummy atoms are used in the smiles representation to highlight cutting points and in the other cases, they are suppressed and hydrogen atoms are added instead. This might change the properties of the fragments with respect to the original molecule as explained in Section 2.

Figure 4a reports the various representations of the fragment benzene resulting from the different parameter settings. In total, 811 benzene fragments are generated from the drug data set using the extendedRECAP fragmentation without dummy atoms. Turning on the flag *x* results in eleven different representations of the benzene structure with one to five cutting points. Additionally, the total number of different fragments increases to 957. This means that some drugs contain more benzene rings with different number and distributions of bonds that were previously mapped to the same fragment and are now separated into their specific fragment patterns. Figure 4b shows the different morpholine structures from the fragmentation. Thereby, 16 fragments were generated from the drug set for both approaches. The figure highlights why the cutting points increase the information about the original compound. Two of the four fragments derived with dummy atoms have cutting points at the nitrogen atom. This information is not available when the flag *x* is turned off which incorrectly increases the number of hydrogen bond donors in these cases. This effect is also illustrated in Figure 4c reporting different sulphonamide fragments from the data set. The 23 sulphonamide structures generated without dummy atoms are separated into four fragments with diverse cutting point patterns. Here, both nitrogen atoms can be part of the bond that was cut or a terminal atom in the original molecule.

### 3.3 Application of weight filter

**Figure 5:**
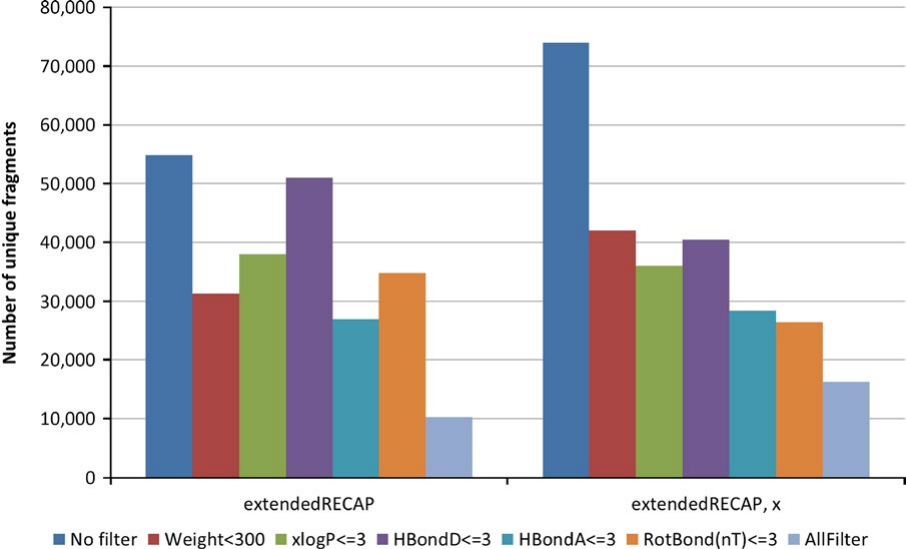
Filtering of generated fragment sets. The application of no filters and rule‐of‐three filters on fragment set generated using exhaustive fragmentation and the extendedRECAP fragmentation rules is compared. The filters include a weight filter <300, ClogP ≤3 (here: *x*logP is used), hydrogen bond donor (HBondD) ≤3, hydrogen bond acceptor (HBondA) ≤3, number of rotatable (non‐terminal) bonds (RotBond(nT)) ≤3 [Colour figure can be viewed at wileyonlinelibrary.com]

In addition to the size filter, the modified molblocks program allows the application of a weight filter to the generated fragments. This could be used to reduce the set of fragments to those fulfilling the “rule‐of‐three.”^14^ The rule‐of‐three proposes that the ideal fragment should fulfil the conditions of a molecular weight <300, ClogP ≤3, the number of hydrogen bond donors and acceptors each ≤3, and the number of rotatable bonds ≤3. In the following, these filters are applied separately and together to analyze the number of unique fragments. The fragments set were generated with exhaustive fragmentation (−*k* 8) using the extendedRECAP method. Figure 5 reports the number of fragments for the sets with and without cutting points. Thereby, the filters have a different effect on the two fragment sets. The application of the weight filter in both cases almost halves the size of the fragment set. When no dummy atoms are used, almost no reduction is obtained applying the hydrogen bond donor filter. The best filter in this case is the hydrogen bond acceptor. However, the size of this set is still more than double of rule‐of‐three compliant fragment set. The reduction in the fragments with cutting points is comparable for the weight and hydrogen bond donor filter and keeps the highest number of unique fragments. The best reduction is achieved applying the rotatable bond filter. Summarizing, the exhaustive fragmentation results in sets of fragments that usually do not comply with the rule‐of‐three. However, the generated sets contain distinct fragments and the key components for bioactivity are generated as analyzed in the following section.

### 3.4 Identification of key fragments

As stated earlier, the FBDD methodology is based on the assumption that fragments are engaged in the same interactions with the target protein even when other parts of the ligands are removed. In this part, we analyze whether those conserved fragments retaining the binding mode can be generated with exhaustive fragmentation. For the analyses, example structures are taken from a recent analysis and study performed by Kozakov et al.^24^ Their study introduces a simple measure to determine how fragments interact with binding energy hot spots to identify the proteins and fragments most suitable for screening. We chose three representative examples from the eight case studies published and tested whether the fragments retaining their initial binding mode were generated with the exhaustive fragmentation. The results are compared to the simple fragmentation approach from the original molblocks programme generating the smallest fragments only, see above. For each example, the best fragments from the simple and exhaustive fragmentation were chosen based on the closest similarity to the key pharmacophore, meaning the fragments were selected to incorporate as many points of interaction with the target as possible (as described by the X‐Ray structure, see below). This was considered as the portion of the molecule driving the molecular recognition event with the target leading to the biological response. Methodologically, the generated fragments were ranked by number of atoms and by the overlap of the smiles strings. The exhaustive fragmentation was able to generate the minimum fragment that retains the correct binding pose.

**Figure 6:**
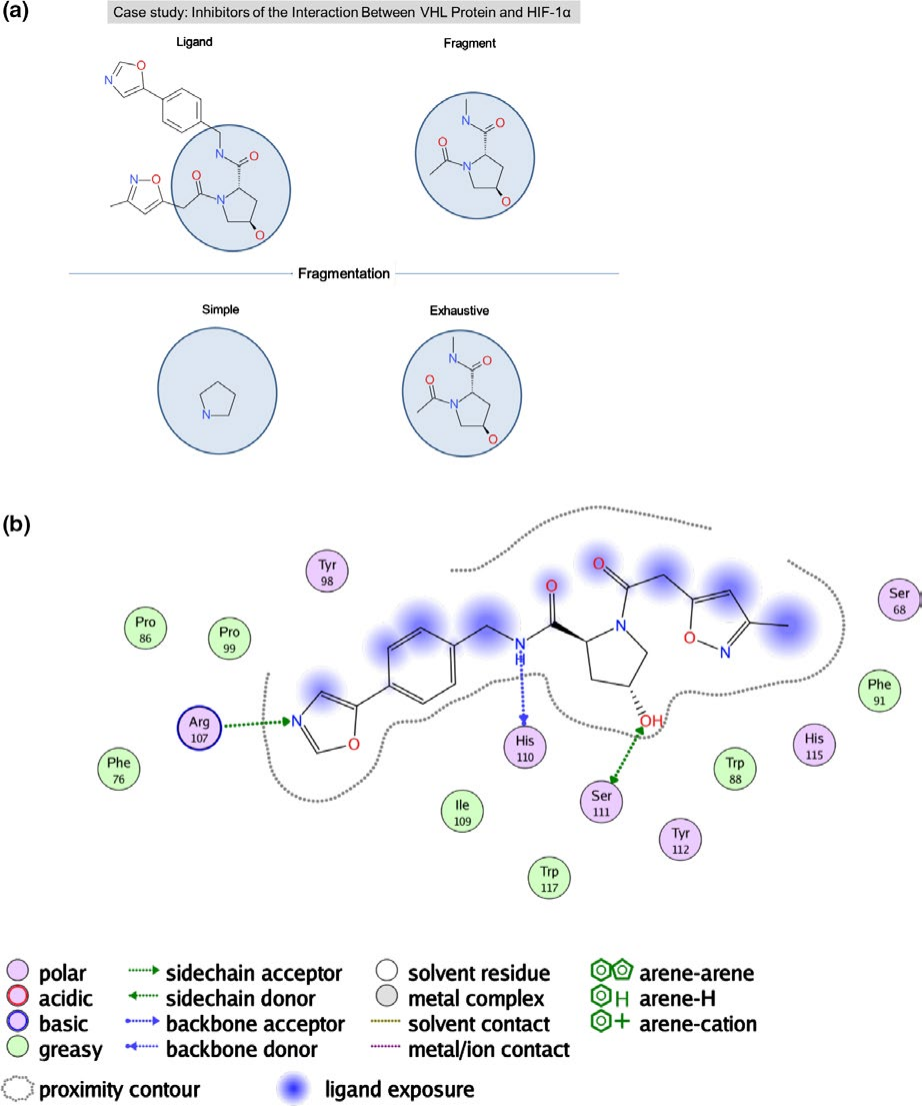
Case study for the identification of key fragments: VHL protein and HIF‐1α. Simple and exhaustive fragmentation are contrasted to analyze how they reproduce conserved fragments from an inhibitor of the interaction between VHL protein and HIF‐1α (PDB code 3ZRC). (a) Fragmentation of the ligand. (b) Key interactions between ligand and protein. The 2D diagrams illustrating the protein–ligand interactions were generated using moe 1 [Colour figure can be viewed at wileyonlinelibrary.com]

Figure 6a,b report the case study of inhibitors of the interaction between VHL protein and HIF‐1α. Figure 6a shows the interaction of the inhibitor with the protein structure obtained from PDB entry 3ZRC. On the top, Figure 6b shows the inhibitor that binds to VHL and blocks its interaction with the transcription factor HIF‐1α. van Molle et al. determined that the fragment *N*‐Hyp on the right binds with a fully conserved position in the main hot spot of VHL.^25^ Applying simple fragmentation using the extendedRECAP method, we can generate a subgroup of the optimal fragment, but not the whole fragment. This is caused by the following two aspects: (i) the simple fragmentation only returns the smallest fragments and (ii) the smallest fragment results from the application of all fragmentation rules in extendedRECAP. This includes, for example a rule cutting the bond of a cyclic amine and any other atom and a rule for breaking the bond of carbon in a ring structure with any atom, resulting in the shown subgroup. However, *N*‐Hyp. can be returned using exhaustive fragmentation due to the combination of several subfragments, that is the smallest fragments are recombined to larger fragments including *N*‐Hyp. This result is representative for other case studies published in Kozakov et al., like for example, the case study of inhibitors of SH2 domain of ^pp60^Src.

**Figure 7:**
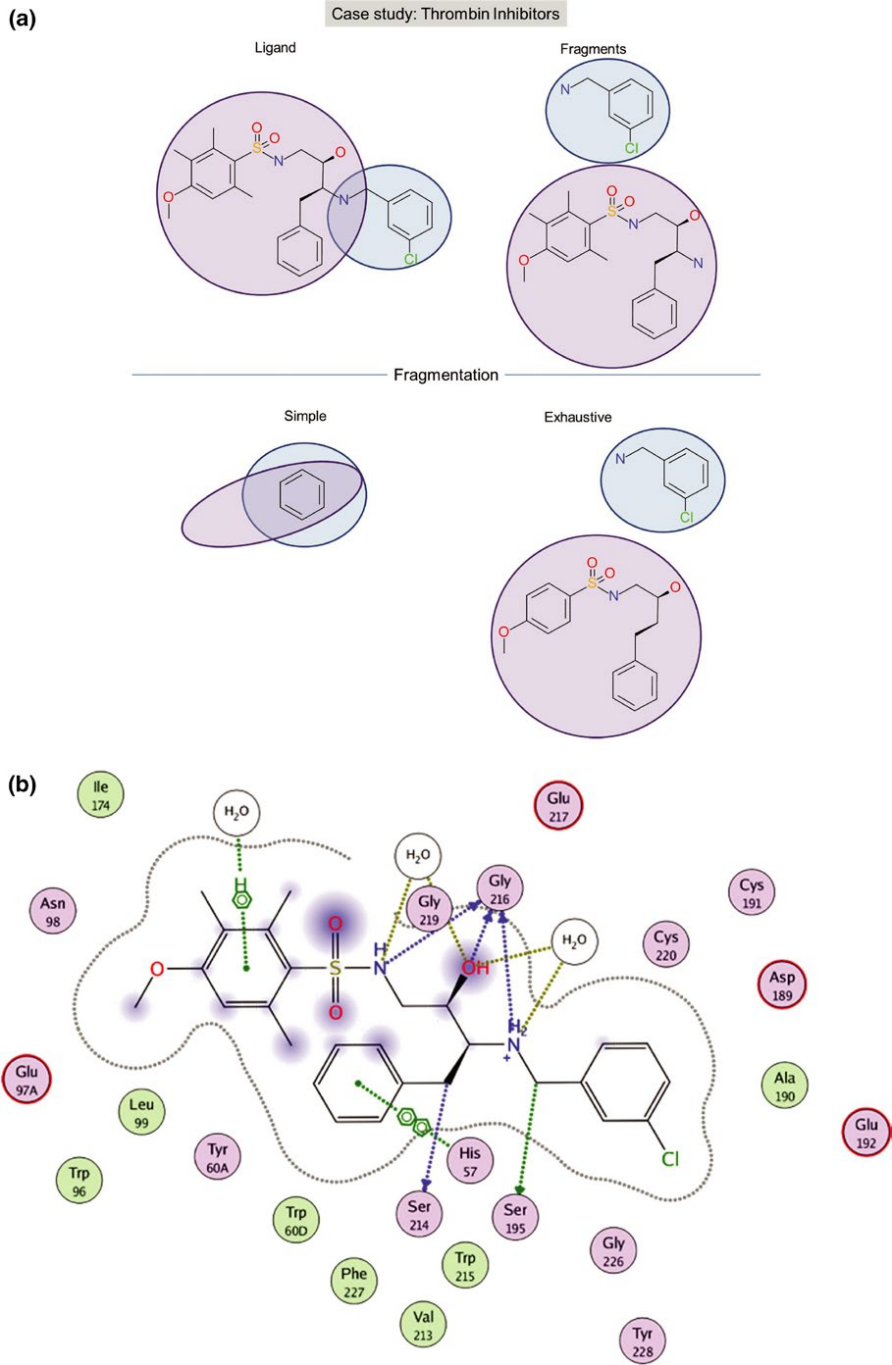
Case studies for the identification of key fragments: thrombin inhibitors. Simple and exhaustive fragmentation are contrasted to analyze how they reproduce conserved fragments from thrombin inhibitors (PDB code 2C8X). (a) Fragmentation of the ligand. (b) Key interactions between ligand and protein. The 2D diagrams illustrating the protein–ligand interactions were generated using moe 1 [Colour figure can be viewed at wileyonlinelibrary.com]

The second case study considered here concerns thrombin inhibitors shown in Figure 7a,b. The inhibitor reported was based on the chlorophenyl fragment on the right top (highlighted in blue). The fragment binds in the same position of the main hot spot in the S1 pocket of thrombin. Again, the simple fragmentation only retrieves a subgroup of this fragment, a benzene ring, whereas the exhaustive fragmentation generates the whole chlorophenyl fragment. The observed behaviour can again be explained by the internal nature of the simple fragmentation approach generating the smallest fragments only and the advantage of the recombination of these fragments in exhaustive fragmentation. The authors also report on a second fragment (highlighted in purple) that binds to the second‐ranked hot spot of thrombin located in the S4 pocket. The benzene ring is again the closest fragment that can be generated with the simple fragmentation. The exhaustive fragmentation is able to almost fully create this fragment. Missing parts include the carbon side chains on the benzene ring and nitrogen. However, the whole fragment can be built by further increasing the combinatorial factor that was set to 8 here. The authors also discuss that this fragment has a molecular weight of 392 and would not be considered a true fragment based on the rule‐of‐three. Therefore, they suggest the use of slightly smaller subfragments of the original fragment structure. These smaller fragments could be generated with the current parameter settings.

**Figure 8:**
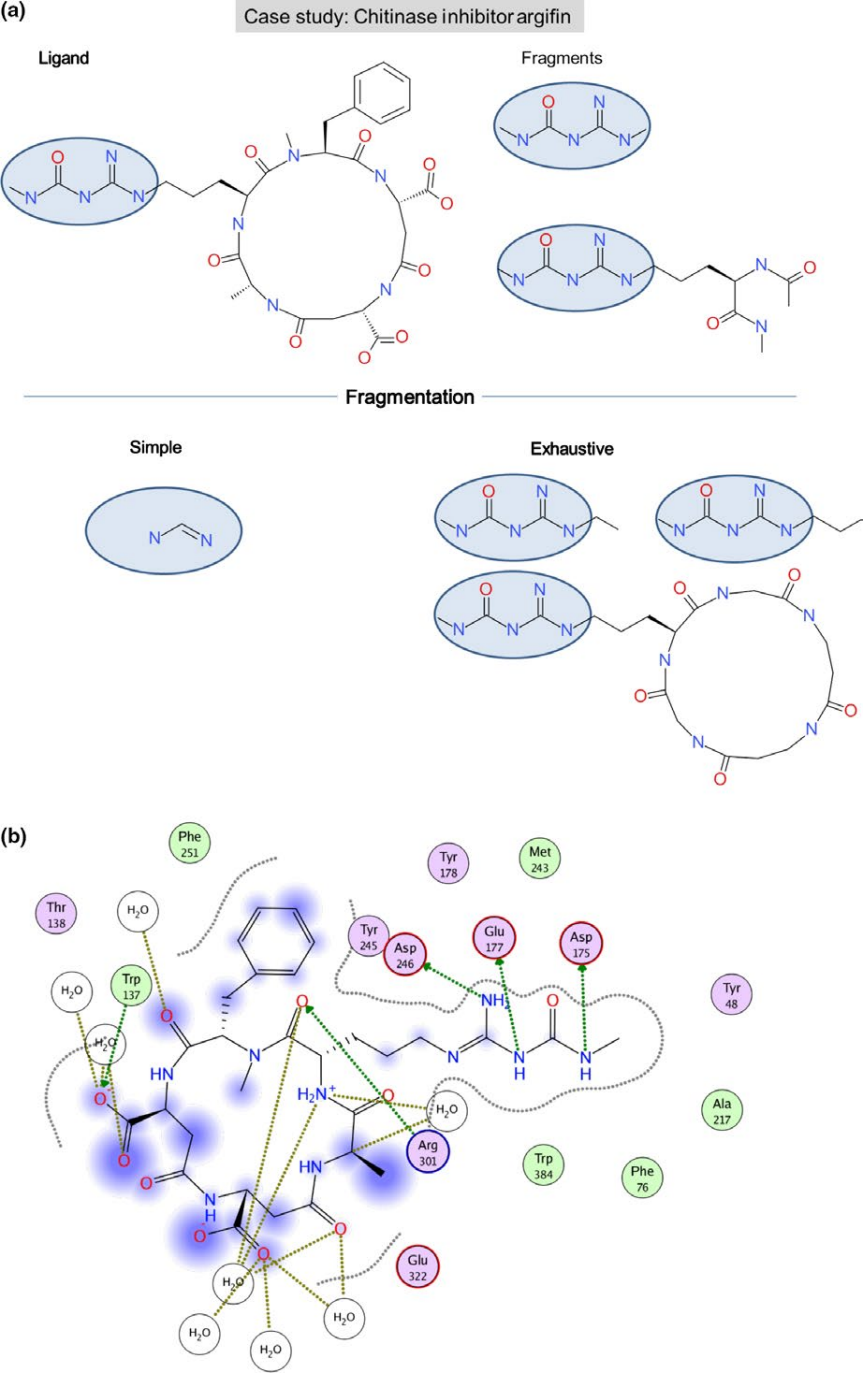
Case studies for the identification of key fragments: the chitinase inhibitor argifin. Simple and exhaustive fragmentation are contrasted to analyze how they reproduce conserved fragments generated from the chitinase inhibitor argifin (PDB code 1W9V). (a) Fragmentation of the ligand. (b) Key interactions between ligand and protein. The 2D diagrams illustrating the protein–ligand interactions were generated using moe 1 [Colour figure can be viewed at wileyonlinelibrary.com]

Finally, Figure 8a,b report on the case study for the chitinase inhibitor argifin. The ligand on the top left in Figure 8b is the natural cyclopentapeptide argifin that inhibits chitinase. The two observed fragments are shorter molecules both containing the dimethylguanylurea moiety. Andersen et al. showed that these two fragments on their own bind in the same position as argifin.^[^ 26 The simple fragmentation is not capable of generating a fragment containing at least five atoms that are a subfragment of argifin. Therefore, in this example, the minimal number of atoms in a fragment was reduced to two to generate a small subgroup of the ligand. The figure also illustrates the three fragments that were generated using exhaustive fragmentation. They are similar to the two fragments described by Andersen et al. One fragment contains the large ring structure as no applied fragmentation rule is able to break the ring. For this, enhanced fragmentation rules have to be defined. Interestingly, the ring structure cannot be cut either utilizing BRICS (data not shown), which highlights the need of other fragmentation rules for ligands in this context.

The three examples show that the modifications of the molecular fragmentation programme molblocks result in fragments that are closer to the key pharmacophores of compounds than those created by other standard fragmentation systems only generating the smallest fragments. The list of generated fragments contains the minimal pharmacophoric elements that are responsible for the interaction of the compound and the biological target. This is the key advantage over other fragmentation approaches.

## 4 CONCLUSIONS

Here, we have presented a modified fragmentation program that can generate an exhaustive sampling of the fragment space associated to a given molecule. We adopted the recently published program suite molblocks and extended its functionality to derive more fragments from a compound structure and not just the smallest, terminal subgroups. We introduced an additional parameter to deal with the capping problem by highlighting the bond cuts in the returned fragments. This extension does not affect the possibility to define new fragmentation rules for different compound spaces as provided by the original program suite. Further introduced parameters allow filtering of the fragment set by their size and molecular weight. The modifications were limited to the fragmentation program from molblocks to keep the functionality of fragment clustering and statistical detection of prominent fragments provided by the analysis method.

The new fragmentation programme was applied to a set of drug compounds to analyze the effect of the modifications on the generated fragments. This exhaustive fragmentation algorithm was able to fragment more drugs being broken down and gave much larger sets of unique fragments. This is because more fragments are derived on a compound level. Furthermore, exhaustive fragmentation yielded additional fragments and fragment frequencies compared to simple fragmentation. The use of dummy atoms to highlight cutting points resulted in fragments with higher information content as previously overlapping fragments can be separated into fragments with different bonding patterns. Although the fragments derived by exhaustive fragmentation did not necessarily fulfil the definition of a true fragment based on the “rule‐of‐three,” the generated set of fragments is diverse and the fragments most suitable for screening can be calculated as opposed to the standard fragmentation.

## CONFLICT OF INTEREST

The authors declare no conflict of interest.

## ENDNOTE

1 Chemical Computing Group Inc., Molecular Operating Environment (moe).
