# Supplementary material for: Exhaustive sampling of the fragment space associated to a molecule leading to the generation of conserved fragments
Source: Chem Biol Drug Des. 2017 Dec 12;91(3):655–67. doi: 10.1111/cbdd.13129 (PMC5836963; doi:10.1111/cbdd.13129)
Supplement: Supplementary file 1 [file CBDD-91-655-s001.pdf]

Exhaustive Sampling of the Fragment Space Associated to a Molecule Leading to the Generation of Conserved Fragments

Kathrin Heikamp, Fabio Zuccotto, Michael Kiczun, Peter Ray, and Ian H. Gilbert*

Drug Discovery Unit, Division of Biological Chemistry and Drug Discovery, School of Life Sciences, University of Dundee, Dundee DD1 5EH, Scotland, United Kingdom

Table S1. Top 100 fragments from BRICS, k=1

Rank	Num	SMILES
1	421	c1ccccc1
2	66	Clc1ccccc1
3	53	Oc1ccccc1
4	51	c1cccnc1
5	40	Fc1ccccc1
6	38	NCC(=O)O
7	38	Cc1ccccc1
8	32	C1CCCCC1
9	28	CCCSC
10	24	CSC(C)(C)C
11	23	Clc1cccc(c1)Cl
12	23	CCCCC
13	23	CCCC=O
14	22	Nc1ccc(cc1)[S](=O)=O
15	22	c1ncc[nH]1
16	21	c1ccc(cc1)Sc1ccccc1
17	19	O=CC(C)C
18	18	Oc1ccccc1O
19	18	OCC(CO)O
20	18	CCC(=O)O
21	17	c1cccs1
22	16	OP(=O)(O)O
23	16	CCOCC
24	15	c1ccncn1
25	15	[O-][N+](=O)c1ccccc1
26	15	c1ccc2c(c1)cccc2
27	15	CCCCC=O
28	15	CCCCC(=O)O
29	14	O=[S](=O)c1ccccc1
30	12	Nc1ccccc1
31	12	c1ccc2c(c1)[nH]cc2
32	12	OC(=O)CCCC(=O)O
33	12	Cc1cccc(c1)C
34	12	C[C@@H](C(=O)O)N
35	11	c1ccc(cc1)Cc1ccccc1
36	11	CCCCCC
37	11	C1CCCC1
38	10	c1ncn[nH]1
39	10	O=CCCCC=O
40	10	CCc1ccc(c(c1)OC)O
41	10	Fc1cccc(c1)F
42	9	Nc1ncnc2c1nc[nH]2
43	9	CCCCO

44	9	c1ccc2c(c1)nccc2
45	9	C[C@@]12CCCC[C@@H]2CCC1
46	9	CCOC=O
47	9	CO/N=C(/c1csc(n1)N)\C=O
48	9	CCNCC
49	9	OCCC=O
50	9	Cc1ccno1
51	9	c1nnn[nH]1
52	9	COC(OC)(C)C
53	8	CCC(C)C
54	8	n1ncn[nH]1
55	8	N#Cc1ccccc1
56	8	CC(=O)CC[C@@]1(C)CCC[C@@H]2[C@@H]1CC[C@]1([C@H]2CCC1)C
57	8	c1ccco1
58	8	CCCCCC(CC)(C)C
59	8	CCCC(=O)O
60	7	CC(F)(F)F
61	7	Brc1ccccc1
62	7	c1ccc2c(c1)[nH]cn2
63	7	CC1CCCCC1
64	7	CCc1c[nH]c2c1cccc2
65	7	c1ccn[nH]1
66	7	Ic1ccccc1
67	7	O=CC(C)(C)C
68	7	CC[C@H](O)C
69	7	CC/N=C(\N)/C
70	7	Cc1cccnc1
71	6	CC(=O)CC[C@@]1(C)CCC[C@@H]2[C@@H]1[C@@H](O)C[C@]1([C@H]2CC[C@H]1O)C
72	6	CCc1cccc(c1)OC
73	6	CC(C(=O)O)C
74	6	c1nccs1
75	6	CCc1ccccc1
76	6	CCCCC[C@H](O)C
77	6	CCN=C(N)N
78	6	O=CCCC(=O)O
79	6	C[C@@H](CO)N
80	6	CCC(=O)C
81	6	NS(=O)(=O)c1ccccc1
82	6	Cn1cnnn1
83	5	C1OCCO1
84	5	O[C@H]1C[C@]2(C)[C@H](O)CC[C@H]2[C@H]2[C@H]1C(C)(C)CCC2
85	5	CCc1ccc(cc1)O
86	5	Fc1cnc2c(c1)c(=O)cc[nH]2
87	5	Nc1cc[nH]c(=O)n1

88	5	Nc1[nH]c(=O)c2c(n1)[nH]cn2
89	5	O[C@@H]1C[C@H](O)c2c(C1)c(O)c1c(c2O)C(=O)c2c(C1=O)cccc2
90	5	Clc1ccccc1Cl
91	5	CC(C(C)C)C
92	5	COCOC
93	5	O[C@H]1C[C@H]([C@@H](C1)C)O
94	5	Nc1nc(=O)c2c([nH]1)[nH]cn2
95	5	C(C#N)C=O
96	5	O=[S](=O)c1ccc(c(c1)S(=O)(=O)N)Cl
97	5	Nc1nc(=O)cc[nH]1
98	5	CCCC(=O)N
99	5	Fc1ccc2c(c1)c(=O)cc[nH]2
100	5	CCCCC[C@H](CCC)C

Table S2. Top 100 fragments from BRICS, k=8

Rank	Num	SMILES
1	421	c1ccccc1
2	166	CCCCC
3	127	Oc1ccccc1
4	125	CCNCC
5	122	CCCCN
6	116	CCCCNC
7	108	Cc1ccccc1
8	88	CCCCCN
9	78	CCCNC
10	72	C1CCCNC1
11	67	Nc1ccccc1
12	66	Clc1ccccc1
13	65	NCC(=O)O
14	62	CCCC=O
15	61	NCCNCC
16	56	CCCN(C)C
17	53	NCC(=O)N
18	52	CCN(C)C
19	51	c1cccnc1
20	49	CCCCCC
21	48	O=Cc1ccccc1
22	46	CNCCN
23	44	CCN(CC)C
24	43	CCCCCNC
25	43	CCNC=O
26	43	N1CCNCC1
27	43	CCNCCC
28	42	COCCO

29	42	O=CNCC(=O)O
30	42	CCOC=O
31	42	CCC(=O)O
32	41	COc1ccccc1
33	40	CCCC(=O)N
34	40	Fc1ccccc1
35	39	CC(=O)NCC(=O)O
36	38	CCCCN(C)C
37	38	NCCN(CC)C
38	37	CNCCNCC
39	36	CCC(C)C
40	35	CCOCC
41	34	NCC(=O)NCC(=O)O
42	34	CCCCCCC
43	33	CN1CCNCC1
44	33	CCC(=O)N
45	32	CCc1ccccc1
46	32	C1CCCCC1
47	32	CCCc1ccccc1
48	32	CCCCC(=O)O
49	32	CCSCCC
50	31	CN1CCCCC1
51	31	O=CC(C)C
52	31	CCCCC=O
53	31	CNC(=O)C
54	30	OCC(O)C
55	30	CCC(CC)O
56	30	CCCSC
57	29	C1CCCN1
58	28	CCCSCCN
59	28	CCCSCCC=O
60	28	OCCNCC
61	28	CCCSC[C@@H](C=O)N
62	27	CCCS[C@@H](NCC(=O)O)CC=O
63	27	O=C[C@H](CSC/C(=C(/C(=O)O)\N)/C)N
64	27	CC1=C(N(CSC1)C(=O)CN)C(=O)O
65	27	O=C[C@H]([C@H]1SCC(=C(N1)C(=O)O)C)N
66	27	O=C1C[C@@H]2N1C(=C(CS2)C)C(=O)O
67	27	CC1=C(N([C@H](SC1)CN)C=O)C(=O)O
68	27	CSC/C(=C(/C(=O)O)\NC(=O)CN)/C
69	27	CSC/C(=C(/C(=O)O)\NC(=O)C)/C
70	27	CC1=C(NCSC1)C(=O)O
71	27	NCCCN
72	27	CC1=C(N[C@H](SC1)CN)C(=O)O
73	27	O=C1NC(=C(C)CSC[C@@H]1N)C(=O)O

74	27	CCCS[C@@H](NCC(=O)O)CN
75	27	CCCS[C@@H](N(CC(=O)O)C=O)CN
76	27	CSC/C(=C(/C(=O)O)\NC=O)/C
77	27	CCCS[C@@H](NCC(=O)O)C
78	27	CCCS[C@@H](N(CC(=O)O)C=O)C
79	27	CCCS[C@@H]1[C@H](N)C(=O)N1CC(=O)O
80	27	CCCS[C@@H]1CC(=O)N1CC(=O)O
81	27	CCCS[C@H]([C@@H](C=O)N)NCC(=O)O
82	27	O=C1[C@@H](N)[C@@H]2N1C(=C(CS2)C)C(=O)O
83	27	CC[C@H](O)C
84	27	CCCSC[C@@H](C(=O)NCC(=O)O)N
85	27	CCCSCCC(=O)NCC(=O)O
86	27	CCCSCNCC(=O)O
87	27	CCCSCN(C(=O)CN)CC(=O)O
88	27	CCCSCN(C(=O)C)CC(=O)O
89	27	CCCSCN(CC(=O)O)C=O
90	27	O=CCCSC/C(=C(/C(=O)O)\N)/C
91	27	C/C(=C(\C(=O)O)/N)/CSC
92	27	O=C1CCSCC(=C(N1)C(=O)O)C
93	27	C[C@H]1SCC(=C(N1)C(=O)O)C
94	27	CCN(CC)CC
95	27	O=CN1[C@@H](C)SCC(=C1C(=O)O)C
96	27	O=CN1CSCC(=C1C(=O)O)C
97	27	C/C(=C(\C(=O)O)/N)/CSCC
98	27	CC(=O)N1CSCC(=C1C(=O)O)C
99	27	O=CC[C@H]1SCC(=C(N1)C(=O)O)C
100	27	C/C(=C(\C(=O)O)/NC=O)/CSCCN

Table S3. Top 100 fragments from BRICS, k=8, x

**Rank**	**Num**	**SMILES**
1	264	*c1ccccc1
2	99	*c1ccc(cc1)*
3	76	*CCN(CC*)*
4	46	*c1ccc(cc1)Cl
5	45	*CCN(CCN(*)*)*
6	42	*c1ccccc1*
7	38	*N1CCN(CC1)*
8	37	OC(=O)C(=*)N(C(=O)*)*
9	37	*N(C(=*)C(=O)O)*
10	35	*Oc1ccc(cc1)*
11	33	O=C(N(C(=*)C(=O)O)*)C(*)*
12	33	*CCCCN(*)*
13	32	CCN(CC)*
14	29	*CCCCC*

15	29	*c1ccc(cc1)F
16	28	*CCC(CC*)*
17	26	*CCN(CC*)C
18	25	OC[C@@H](OC(*)*)*
19	25	*c1ccccn1
20	24	*Cc1ccc(cc1)*
21	24	*N(C(=*)C(=O)O)C(=O)[C@@H](N*)*
22	24	*C(CC(*)*)CC(*)*
23	24	*C(SC(C(*)*)(C)C)*
24	24	*N(CCN(C)*)*
25	23	*C1CCCCC1
26	23	*c1ccc(cc1)O
27	23	*c1ccc(cc1)C(=O)*
28	23	*OCC(C*)O
29	22	*C(=O)N1[C@@H](*)SC([C@@H]1*)(C)C
30	22	*C(=O)N([C@H](SC(C(*)*)(C)C)*)*
31	22	*CC(=*)CSC(*)*
32	22	*C(SC([C@@H](N(C(=O)*)*)*)(C)C)*
33	22	*C(SC([C@@H](N(*)*)*)(C)C)*
34	22	*CCN(CCN(*)*)C
35	22	*CCN(CCN(C)*)*
36	22	*N([C@H](SC(C(*)*)(C)C)*)*
37	22	Nc1ccc(cc1)S(=O)(=O)*
38	21	*N1CCN(CC1)C
39	21	*Cc1ccccc1
40	21	*C([C@H](N(C(=O)*)*)SC(C(*)*)(C)C)*
41	21	*c1ccc(c(c1)*)*
42	21	*C([C@H](N(*)*)SC(C(*)*)(C)C)*
43	20	*C(SC([C@@H](N(C(=O)*)*)C(=O)O)(C)C)*
44	20	*CCCCCN(*)*
45	20	*C(=O)NC(=O)*
46	20	*N[C@@H]1C(=O)N([C@@H]1SC(C(*)*)(C)C)*
47	20	*NC(C)(C)C
48	20	*C(SC([C@@H](N(C(=O)C(*)*)*)*)(C)C)*
49	20	*C([C@@H](SC([C@@H](N(C(=O)*)*)*)(C)C)*)*
50	20	*N[C@@H]([C@H](N(*)*)SC(C(*)*)(C)C)C(=O)*
51	20	*CCCCN(C*)*
52	20	*C(C(=O)N1[C@@H](*)SC([C@@H]1*)(C)C)*
53	20	*C(SC([C@H](C(=O)O)N(*)*)(C)C)*
54	20	*N([C@@H]([C@@H](C(=O)*)*)SC(C(*)*)(C)C)*
55	20	*C([C@H]1SC([C@@H](N1C(=O)*)*)(C)C)*
56	20	*C(C(=O)N([C@H](SC(C(*)*)(C)C)*)*)*
57	20	*C([C@H]1SC([C@@H](N1*)*)(C)C)*
58	19	*C/C(=C(\C(=O)O)/N(*)*)/CSC(*)*
59	19	*C(=O)N1[C@@H](*)SC([C@@H]1C(=O)O)(C)C

60	19	*[C@H]1SC([C@@H](N1*)C(=O)O)(C)C
61	19	*C1CCN(CC1)*
62	19	*N[C@@H]1C(=O)N2[C@@H]1SC([C@@H]2*)(C)C
63	19	*CC1=C(C(=O)O)N([C@H](SC1)*)C(=O)*
64	19	*N[C@@H]([C@H]1SC([C@@H](N1*)*)(C)C)C(=O)*
65	19	*CC1=C(C(=O)O)N([C@H](SC1)*)*
66	19	*C([C@H]1SC([C@@H](N1*)C(=O)O)(C)C)*
67	19	*C([C@H]1SC([C@@H](N1C(=O)*)C(=O)O)(C)C)*
68	19	*CC(CCN(*)*)*
69	19	*CC(CCN(C*)*)*
70	19	*CCN(C)C
71	19	*N[C@H]1[C@H](*)SC([C@@H](N(C1=O)*)*)(C)C
72	19	*N1CCCCC1
73	19	*NCC(CO*)O
74	19	*NCC(C*)O
75	19	*CCC(CCN(*)*)*
76	19	*c1cccc(c1)*
77	19	*N[C@@H]([C@@H](SC([C@@H](N(*)*)*)(C)C)*)C(=O)*
78	19	*OC(CC(*)*)CC(*)*
79	19	*N[C@@H]([C@@H](SC(C(*)*)(C)C)*)C(=O)*
80	19	*C/C(=C(/N(C(=O)*)*)\C(=O)O)/CSC(*)*
81	19	*C(SC([C@@H](N(C(=O)C(*)*)*)C(=O)O)(C)C)*
82	19	*N[C@H](C(=O)N(*)*)[C@@H](SC(C(*)*)(C)C)*
83	18	*N[C@@H]([C@@H](SCC(=*)C*)*)C(=O)*
84	18	*CCOCC*
85	18	OC(=O)[C@@H]1N([C@H](SC1(C)C)*)C(=O)C(*)*
86	18	*Nc1ccc(cc1)*
87	18	*c1ccc(c(c1)O)O
88	18	*CC(=*)CS[C@@H](N(C(=*)C(=O)O)C(=O)*)*
89	18	*OC(=O)CC
90	18	*N[C@@H]([C@H]1SC([C@@H](N1*)C(=O)O)(C)C)C(=O)*
91	18	*N[C@@H]1C(=O)N2[C@@H]1SC([C@@H]2C(=O)O)(C)C
92	18	*CC(=*)CS[C@@H](N(C(=*)C(=O)O)*)*
93	17	*N[C@H]1[C@H](*)SC([C@@H](N(C1=O)*)C(=O)O)(C)C
94	17	*C([C@@H](SC([C@H](C(=O)O)N(*)*)(C)C)*)*
95	17	OC(=O)[C@@H](C(S[C@H](C(*)*)*)(C)C)N(C(=O)*)*
96	17	*C(CCCN(*)*)*
97	17	*C(c1ccccc1)*
98	17	*CCN(C*)C*
99	17	*NS(=O)(=O)c1ccc(cc1)N
100	17	*N[C@H]([C@@H](SCC(=*)C*)*)*

Table S4. Top 100 fragments from CCQ, k=1

Rank	Num	SMILES

1	49	Cc1ccccc1
2	48	CCCCC
3	46	O=CN(C)C
4	45	C[N+](C)(C)C
5	28	COCOC
6	26	CSCN(C=O)C=C
7	23	CN(C=O)CSC
8	21	CCc1ccccc1
9	20	OCc1ccccc1
10	18	c1cccnc1
11	17	Nc1nccs1
12	15	Cn1cncc1
13	15	C1CCCCC1
14	15	COc1ccc(cc1)C
15	14	c1cccs1
16	13	COC(=O)N
17	13	CCC[C@H](C(C)C)CCCC1=CC(=O)C=C[C@H]1C
18	13	COP(=O)(O)O
19	11	CCCCCCC
20	11	C[C@@H]1C=CC(=O)C=C1
21	10	CCCc1ccccc1
22	10	OCc1ccc(c(c1)O)O
23	10	O=CNC(=O)NC=O
24	10	O/C=C/C=O
25	9	c1ccco1
26	9	CC[C@@H]([C@@H]1CCC2=CC(=O)CC[C@@]2(C1)C)C(C)C
27	9	CN/C=N\C
28	8	Cc1ccc(cc1)O
29	8	O=CNc1c(C)cccc1C
30	8	CSc1nnnn1C
31	8	c1ncc[nH]1
32	8	COc1ccccc1OC
33	7	COCSC
34	7	OC(c1ccccc1)c1ccccc1
35	7	CNC(=O)OC
36	7	CC[C@@H]([C@@H]1CCC2=CC(=O)C=C[C@@]2(C1)C)C(C)C
37	7	O=CNC=O
38	7	CC[C@@H]1[C@H](C)CC[C@H]2[C@H]1CCC1=CC(=O)CC[C@]21C
39	7	NCc1ccccc1
40	6	CN(Cc1ccccc1)C
41	6	CCC[C@H](C(C)C)CC
42	6	COc1ccccc1
43	6	CCCCCC
44	6	OC(P(=O)(O)O)P(=O)(O)O
45	6	Cc1ccc(c(c1)O)O

46	6	CNC(=O)NS(=O)(=O)c1ccc(cc1)C
47	6	CCC(C)C
48	6	CCc1c(C)ccc(c1OC)O
49	6	CN1c2ccccc2Sc2c1cccc2
50	5	CN=C(N)N
51	5	COCc1ccccc1
52	5	CCCCC[C@H](CCC)C
53	5	Cc1c[nH]c2c1cccc2
54	5	CNc1ccc(cc1)C(=O)NC
55	5	CN(C=O)/C=C/SC
56	5	CN1c2cc(ccc2Sc2c1cccc2)C(F)(F)F
57	5	CN(C(c1ccccc1)c1ccc(cc1)Cl)C
58	5	CCCCCCCCCCC
59	5	CCCCCCCCCCCCCCC
60	5	C[n+]1ccccc1
61	5	COCn1cnc2c1ncnc2N
62	5	COc1ccncc1C
63	5	CCC/C=C\CCCC=C
64	5	c1nccs1
65	5	[O-][N+](=O)c1ccco1
66	5	CNC(=N)N
67	5	CC([C@H](CCCC1=CC(=O)C=C[C@H]1C)C)C
68	5	CC(c1ccccc1)C
69	4	CC(c1ccccc1)c1ccccc1
70	4	COCOCc1c(C)c(O)c2c(c1O)C(=O)c1c(C2=O)cccc1OC
71	4	O=CN(C=O)C
72	4	NCc1ccc(cc1)O
73	4	COc1c(N(C)C)c(F)cc2c1n(C)cc(c2=O)C(=O)O
74	4	O=CN(c1ccccc1)C
75	4	CCCc1cccc2c1c(C)c[nH]2
76	4	O=Cc1ccccc1
77	4	C=Cc1ccccc1
78	4	COC(c1ccccc1)c1ccccc1
79	4	CC[C@H](C(C)C)CCCC1=CC(=O)CC[C@H]1C
80	4	CCC(CC)C
81	4	CN1c2cc(ccc2Sc2c1cccc2)C(=O)C
82	4	COc1c(C)cncc1C
83	4	COc1cc2nc(nc(c2cc1OC)N)N(C)C
84	4	CCC1CCCC1
85	4	COC(=O)c1ccccc1
86	4	CN(C(=O)NC=O)N=C
87	4	OCc1ccc(c(c1)CO)O
88	4	C[C@@H]1[C@H](C)CC[C@H]2[C@H]1CC[C@H](C2(C)C)C
89	4	CS(=O)(=O)O
90	4	n1ccncc1

91	4	Cn1cnnc1
92	4	COCNC=O
93	4	CC([C@H]([C@@H]1CCC2=CC(=O)C=C[C@@]2(C1)C)C)C
94	4	CCCC(C)C
95	4	CC1CCCCC1
96	4	CN(C(=O)NC)N=O
97	4	O=COCOC=O
98	4	CC[C@@H]1[C@H](C)CC[C@H]2[C@H]1CCc1c2ccc(c1)O
99	4	COc1ccc(cc1)Cl
100	4	Cn1cncn1

Table S5. Top 100 fragments from CCQ, k=8

Rank	Num	SMILES
1	137	CCN(C)C
2	64	CCOC=O
3	59	CCN(CC)C
4	51	CCCN(C)C
5	50	COC(=O)C
6	49	Cc1ccccc1
7	48	CCCCC
8	46	O=CN(C)C
9	45	C[N+](C)(C)C
10	40	CNC(C)C
11	39	CCCCN(C)C
12	37	NCC(=O)O
13	35	CNCCO
14	34	CN1CCCCC1
15	34	O=COCC=O
16	28	CN(CCN(C)C)C
17	28	COCOC
18	27	O=COC(C)C
19	26	CCCN(CC)C
20	26	OCC(CO)O
21	26	CCC(=O)O
22	26	C1SCC=CN1C=O
23	26	CSCN(C=O)C=C
24	24	OCCCO
25	24	CN1CCCC1
26	23	O=CN1CSC(C1)C
27	23	OCCNCC
28	23	O=CN1CSC(C1)(C)C
29	23	O=CN(CSC(C)C)C
30	23	OCCN(C)C
31	23	O=CN1CSCC1

32	23	CN(C=O)CSC
33	23	CN(C=O)CSCC
34	22	CSCN(C(=O)CNC=O)C=C
35	22	O=CN[C@@H]1C(=O)N2[C@@H]1SCC=C2
36	22	O=CNC[C@H](N(C=O)C=C)SC
37	22	O=CN[C@@H]1C(=O)N([C@@H]1SC)C=C
38	22	C(C(=O)N1CSCC=C1)NC=O
39	22	O=CNC[C@H]1SCC=CN1C=O
40	21	O=CN1CSCC=C1C(=O)O
41	21	OCCNC(C)C
42	21	CCC(=O)OC
43	21	CCc1ccccc1
44	21	CSCN(C(=C)C(=O)O)C=O
45	20	O=CN1CSC([C@@H]1C(=O)O)C
46	20	O=CN1CSC[C@@H]1C(=O)O
47	20	CSCN(CC(=O)O)C=O
48	20	O=CN1CSC([C@@H]1C(=O)O)(C)C
49	20	OCc1ccccc1
50	20	CCNC=O
51	20	O=CN(CC(=O)O)CSC(C)C
52	20	CCSCN(C=O)CC(=O)O
53	20	CC[N+](C)(C)C
54	19	COCCN(C)C
55	18	c1cccnc1
56	18	CSCN(C(=C)C(=O)O)C(=O)CNC=O
57	18	CC[N+](CC)(C)C
58	18	O=CNC[C@H]1SCC=C(N1C=O)C(=O)O
59	18	O=CN[C@@H]1C(=O)N2[C@@H]1SCC=C2C(=O)O
60	18	O=CNCC(=O)N1CSCC=C1C(=O)O
61	18	O=CNC[C@H](N(C(=C)C(=O)O)C=O)SC
62	18	O=CN[C@@H]1C(=O)N([C@@H]1SC)C(=C)C(=O)O
63	18	CN1CCN(CC1)C
64	17	CNC(C)(C)C
65	17	Nc1nccs1
66	17	OCC(=O)CO
67	17	OCC(=O)COC=O
68	15	CNC(=O)C
69	15	COc1ccc(cc1)C
70	15	Cn1cncc1
71	15	C1OCCO1
72	15	C1CCCCC1
73	15	O=COCC[N+](C)(C)C
74	15	CO/N=C\C(=O)NC
75	14	O=CNCC(=O)N(CSC(C)C)C
76	14	c1cccs1

77	14	O=CNC[C@H]1SC(CN1C=O)C
78	14	O=CN[C@@H]1C(=O)N2[C@@H]1SC(C2)(C)C
79	14	O=CNC[C@H]1SCCN1C=O
80	14	O=CN[C@@H]1C(=O)N([C@@H]1SC(C)C)C
81	14	O=CNCC(=O)N1CSC(C1)(C)C
82	14	CN(C(=O)CNC=O)CSC
83	14	O=CNCC(=O)N1CSC(C1)C
84	14	O=CN[C@@H]1C(=O)N2[C@@H]1SC(C2)C
85	14	O=CNC[C@H]1SC(CN1C=O)(C)C
86	14	O=CNC[C@H](N(C=O)C)SC(C)C
87	14	O=CNC[C@H](N(C=O)C)SC
88	14	O=CNC[C@H](N(C=O)C)SCC
89	14	O=CN[C@@H]1C(=O)N([C@@H]1SCC)C
90	14	O=CN[C@@H]1C(=O)N([C@@H]1SC)C
91	14	O=CN[C@@H]1C(=O)N2[C@@H]1SCC2
92	14	O=CNCC(=O)N(CSCC)C
93	14	O=CNCC(=O)N1CSCC1
94	13	COP(=O)(O)O
95	13	CCC[C@H](C(C)C)CCCC1=CC(=O)C=C[C@H]1C
96	13	COC(=O)N
97	13	O=COCCC[N+](C)(C)C
98	13	CCC[C@H]([C@@H](CCO)C)CCCC1=CC(=O)C=C[C@H]1C
99	12	O=COCCC[N+](CC)(C)C
100	12	O=CNC[C@H]1SC([C@@H](N1C=O)C(=O)O)(C)C

Table S6. Top 100 fragments from CCQ, k=8, x

Rank	Num	SMILES
1	48	*CCN(C*)C*
2	34	*Cc1ccccc1
3	28	*C(=O)OCC
4	26	*C(=O)OCC(=O)*
5	25	*CN(CC)C*
6	24	*[C@H](C(=O)O)N
7	23	*C(=O)N1[C@@H](*)SCC(=C1*)*
8	23	*CS[C@@H](N(C(=C(*)*)*)C(=O)*)*
9	23	*CCC(=O)O
10	22	*C(N([C@H](SC(C)(*)*)*)C(=O)*)*
11	22	*C(N([C@H](SC(*)(*)*)*)C(=O)*)*
12	22	*C(N([C@H](SC(C)(C)*)*)C(=O)*)*
13	22	*C(=O)N1[C@@H](*)SC([C@@H]1*)(*)*
14	22	*C(=O)N1[C@@H](*)SC([C@@H]1*)(*)C
15	22	*C(=O)N1[C@@H](*)SC([C@@H]1*)(C)C
16	21	*CN(CC)CC
17	20	*C(=O)N[C@@H]1C(=O)N2[C@@H]1SCC(=C2*)*

18	20	*COC(=O)C
19	20	*CN(C(=O)*)C*
20	20	*CS[C@@H]1[C@H](NC(=O)*)C(=O)N1C(=C(*)*)*
21	19	*C(=O)N1[C@@H](*)SC([C@@H]1C(=O)O)(C)C
22	19	*C(=O)N1[C@@H](*)SC([C@@H]1C(=O)O)(*)C
23	19	*C(=O)N1[C@@H](*)SCC(=C1C(=O)O)*
24	19	OC(=O)[C@H](N([C@H](SC(*)(*)*)*)C(=O)*)*
25	19	OC(=O)[C@H](N([C@H](SC(C)(*)*)*)C(=O)*)*
26	19	OC(=O)[C@H](N([C@H](SC(C)(C)*)*)C(=O)*)*
27	19	*CCN(CC*)C*
28	19	*C(=O)N[C@H]([C@H]1SCC(=C(N1C(=O)*)*)*)*
29	19	*CN(CCN(C*)C*)C*
30	19	*C(=O)N1[C@@H](*)SC([C@@H]1C(=O)O)(*)*
31	18	*CNC(C)C
32	18	*CS[C@@H](N(C(=C(*)*)C(=O)O)C(=O)*)*
33	18	*CS[C@@H](N(C(=C(*)*)*)C(=O)*)[C@@H](NC(=O)*)*
34	18	*CN(C(=O)*)C(*)*
35	18	*C(=O)N[C@H](C(=O)N1[C@@H](*)SCC(=C1*)*)*
36	17	*CCCCC*
37	17	*c1ccccn1
38	17	OCC(=O)C(O)(*)*
39	17	*CNC(C)(C)*
40	16	*c1csc(n1)N
41	16	*CNC(C)(C)C
42	16	*C(=O)N[C@@H]1C(=O)N2[C@@H]1SCC(=C2C(=O)O)*
43	16	*CS[C@@H]1[C@H](NC(=O)*)C(=O)N1C(=C(*)*)C(=O)O
44	16	*CC(OC(=O)*)C*
45	15	*C(c1ccccc1)*
46	15	*CS[C@@H](N(C(=C(*)*)C(=O)O)C(=O)*)[C@@H](NC(=O)*)*
47	15	*C(=O)N[C@H]([C@H]1SCC(=C(N1C(=O)*)C(=O)O)*)*
48	15	*CCc1ccccc1
49	15	*C(=O)N[C@H](C(=O)N1[C@@H](*)SCC(=C1C(=O)O)*)*
50	15	*C(OC(=O)C)*
51	14	*C(=O)N[C@@H]1C(=O)N2[C@@H]1SC([C@@H]2*)(*)C
52	14	*C(=O)OC(C(=O)*)(*)*
53	14	*CS[C@@H](N(C(=C(*)*)*)C(=O)[C@@H](NC(=O)*)*)*
54	14	*CCCN(C*)C*
55	14	*C(=O)N[C@@H]1C(=O)N2[C@@H]1SC([C@@H]2*)(C)C
56	14	*C(=O)N[C@@H]1C(=O)N2[C@@H]1SC([C@@H]2*)(*)*
57	14	*C(=O)N[C@@H]1C(=O)N([C@@H]1SC(C)(C)*)C(*)*
58	14	*C(=O)N[C@@H]1C(=O)N([C@@H]1SC(C)(*)*)C(*)*
59	14	*C(=O)N[C@@H]1C(=O)N([C@@H]1SC(*)(*)*)C(*)*
60	13	*C(=O)OCC(=O)C(O)(*)*
61	13	*COCCN(C*)C*
62	13	*c1cccs1

63	13	*C[C@@H](OC(=O)*)*
64	12	*C(=O)N[C@H]([C@H](N(C(=O)*)C(*)*)SC(C)(C)*)*
65	12	*C(=O)N[C@@H]1C(=O)N([C@@H]1SC(C)(C)*)[C@@H](C(=O)O)*
66	12	*C(NCC(O)*)*
67	12	*C(NCC(O)*)C
68	12	*C(CNC(C)C)O
69	12	*Cn1cncc1
70	12	*C(=O)N[C@@H]1C(=O)N2[C@@H]1SC([C@@H]2C(=O)O)(*)*
71	12	*C(=O)N[C@@H]1C(=O)N([C@@H]1SC(C)(*)*)[C@@H](C(=O)O)*
72	12	*C(=O)N[C@H]([C@H]1SC([C@@H](N1C(=O)*)*)(C)C)*
73	12	*C(=O)N[C@H]([C@H]1SC([C@@H](N1C(=O)*)*)(*)C)*
74	12	*C(=O)N[C@@H]1C(=O)N2[C@@H]1SC([C@@H]2C(=O)O)(*)C
75	12	*C(=O)N[C@H]([C@H]1SC([C@@H](N1C(=O)*)*)(*)*)*
76	12	*C(=O)N[C@@H]1C(=O)N2[C@@H]1SC([C@@H]2C(=O)O)(C)C
77	12	*C(=O)N[C@@H]1C(=O)N([C@@H]1SC(*)(*)*)[C@@H](C(=O)O)*
78	12	*C(=O)N[C@H]([C@H](N(C(=O)*)C(*)*)SC(C)(*)*)*
79	12	*C(=O)N[C@H](C(=O)N1[C@@H](*)SC([C@@H]1*)(*)*)*
80	12	*C(=O)N[C@H](C(=O)N([C@H](SC(C)(C)*)*)C(*)*)*
81	12	*C[N+](C)(C)C
82	12	*C(=O)N[C@H](C(=O)N1[C@@H](*)SC([C@@H]1*)(*)C)*
83	12	*C(=O)N[C@H](C(=O)N([C@H](SC(C)(*)*)*)C(*)*)*
84	12	*C(=O)N[C@H](C(=O)N([C@H](SC(*)(*)*)*)C(*)*)*
85	12	*C(=O)N[C@H](C(=O)N1[C@@H](*)SC([C@@H]1*)(C)C)*
86	12	*C(=O)N[C@H]([C@H](N(C(=O)*)C(*)*)SC(*)(*)*)*
87	12	*C1CCCCC1
88	11	CC(=O)OC(*)(*)*
89	11	*CCCN(C(=O)*)C(*)*
90	11	*CS[C@@H](N(C(=C(*)*)C(=O)O)C(=O)[C@@H](NC(=O)*)*)*
91	11	*C(=O)N[C@H](C(=O)N1[C@@H](*)SC([C@@H]1C(=O)O)(*)C)*
92	11	O[C@H]([C@H](O)*)[C@@H](O)*
93	11	*C(=O)N[C@H]([C@H]1SC([C@@H](N1C(=O)*)C(=O)O)(*)*)*
94	11	*C(=O)N[C@H](C(=O)N1[C@@H](*)SC([C@@H]1C(=O)O)(C)C)*
95	11	*C(=O)N[C@H]([C@H](N([C@@H](C(=O)O)*)C(=O)*)SC(*)(*)*)*
96	11	*C(OC(=O)*)C
97	11	*COC(=O)N
98	11	*C(=O)N[C@H]([C@H](N([C@@H](C(=O)O)*)C(=O)*)SC(C)(C)*)*
99	11	*C(=O)N[C@H](C(=O)N([C@@H](C(=O)O)*)[C@H](SC(C)(C)*)*)*
100	11	*C(=O)N[C@H](C(=O)N1[C@@H](*)SC([C@@H]1C(=O)O)(*)*)*

Table S7. Top 100 fragments from REACP, k=1

Rank	Num	SMILES
1	85	Cc1ccccc1
2	67	c1ccccc1
3	63	N1CCNCC1

4	39	C1CCCNC1
5	38	CCCCC
6	26	O=[S](=O)c1ccccc1
7	25	CCc1ccccc1
8	22	CCCc1ccccc1
9	20	N[C@@H]1CN2[C@@H]1SC([C@@H]2C)(C)C
10	17	c1cccnc1
11	17	CCCCc1ccccc1
12	16	c1ncc[nH]1
13	16	Fc1ccccc1
14	15	c1ccc(cc1)Cc1ccccc1
15	14	C1CNCCO1
16	13	Clc1ccccc1
17	12	CCCCCC
18	12	c1ncc2c(n1)[nH]cn2
19	11	Oc1ccccc1
20	11	Cc1ccccc1O
21	10	N[C@@H]1CN2[C@@H]1SCC(=C2C)C
22	10	C1CCCN1
23	9	Nc1ccccc1
24	9	C/C(=N\OC)/c1cscn1
25	9	Cc1ccc(cc1)Cl
26	9	n1ncn[nH]1
27	9	Clc1ccc(cc1)Cc1ccccc1
28	8	Cc1cccc(c1N)C
29	8	CCC[C@H](N)C
30	8	c1ccncn1
31	8	c1ncn[nH]1
32	8	O=c1[nH]c(=O)c2c([nH]1)nc[nH]2
33	8	Cc1ccc(cc1)C
34	8	c1ccc2c(c1)ncnc2
35	8	CC1=C(C)C(CCC1)(C)C
36	8	OCC[C@@]1(O)CC[C@@H]2[C@]1(C)C[C@H](O)[C@H]1[C@H]2CCC2=CCCC[C@]12C
37	7	CCNCC
38	7	c1ccc2c(c1)Sc1c(N2)cccc1
39	7	CC(C)(C)C
40	7	CC1=C(CO)CS[C@H]2N1C[C@H]2N
41	7	C[C@@H]1CCCN1
42	7	CCC(C)C
43	6	CCCCC[C@H](O)C
44	6	CC(F)(F)F
45	6	N[C@@H]1CN2[C@@H]1SCC(=C2C)CSc1nnn[nH]1
46	6	O=c1[nH]cnc2c1nc[nH]2
47	6	Clc1cccc(c1)Cl

48	6	OC[C@H]1OCC[C@@H]1O
49	6	Cc1ccco1
50	6	O=c1nc[nH]c2c1nc[nH]2
51	5	CCc1ccc(cc1)O
52	5	CC([C@H]1CC[C@@H]2[C@]1(C)CCCC2)C
53	5	CCc1ccc(cc1Cl)Cl
54	5	OCC[C@@]1(O)[C@@H](C)C[C@@H]2[C@]1(C)C[C@H](O)[C@]1([C@H]2CCC2=CCC=C[C@]12C)F
55	5	Fc1cnc2c(c1)c(=O)c(c[nH]2)C
56	5	FC(c1ccc2c(c1)Nc1c(S2)cccc1)(F)F
57	5	Fc1ccc2c(c1)c(=O)c(c[nH]2)C
58	5	CC(c1ccc(c(c1)O)O)O
59	5	CC[C@H]1[C@@H](O)C[C@H]([C@@H]1C)O
60	5	Cc1cccnc1
61	5	Cc1ccccc1C
62	5	FC(c1ccccc1)(F)F
63	5	NC1CCCCC1
64	5	Cc1cccc(c1)Cl
65	5	Cc1oncc1C
66	5	C[C@H]1CN[C@H]2C(=C1)c1cccc3c1c(C2)c[nH]3
67	5	OC[C@H](c1ccccc1)C
68	5	O[C@H]1CC[C@@H]2[C@]1(C)CC[C@H]1[C@H]2CCC2=CCCC[C@]12C
69	5	CCc1cccs1
70	5	CCCc1ccc(cc1)O
71	5	O[C@@H]1CCC[C@H](C1)O
72	5	NCCc1ccc(cc1)[S](=O)=O
73	5	CC(Cc1ccccc1)C
74	5	Cc1cccc2c1cccc2
75	5	Cc1ccccn1
76	5	O=c1nccc[nH]1
77	5	c1nnn[nH]1
78	5	OCC(O)C
79	4	CC(c1ccccc1)(c1ccccc1)O
80	4	C[C@@H](c1ccccc1)O
81	4	C1CCCCC1
82	4	CCC(c1ccccc1)(c1ccccc1)C
83	4	c1ccc2c(c1)[nH]cn2
84	4	Cc1cccc(c1)C
85	4	CCCCCCCCCC
86	4	OC1CCCCC1
87	4	O[C@@H]1C[C@@H]2CC[C@H](C1)[NH2+]2
88	4	Oc1ccc2c3c1O[C@@H]1[C@@]43CCN[C@H](C2)[C@]4(O)CCC1
89	4	CC1(CCNCC1)c1ccccc1
90	4	O[C@@H]1CC[C@]2([C@H](C1)CC[C@@H]1[C@@H]2CC[C@]2([C@H]1CC[C@@H]2O)C)C
91	4	OCC(c1ccccc1)C

92	4	CC[C@@H](C[C@H](O)C)O
93	4	Oc1cc(cc(c1)O)C(O)C
94	4	O=c1cc[nH]c(=O)[nH]1
95	4	CC(c1ccccc1)(C1CCCCC1)O
96	4	Cc1ccc(cc1)[S](=O)=O
97	4	OC[C@H]1OC[C@@H]([C@@H]1O)O
98	4	OCC[C@@]1(O)CC[C@@H]2[C@]1(C)C[C@H](O)[C@H]1[C@H]2CCC2=CCC=C[C@]12C
99	4	CCc1ccc2c(c1)Nc1c(S2)cccc1
100	4	CC1=C(C)C(C(=C(N1)C)C)c1cccc(c1)[N+](=O)[O-]

Table S8. Top 100 fragments from REACP, k=8

Rank	Num	SMILES
1	85	Cc1ccccc1
2	67	c1ccccc1
3	63	N1CCNCC1
4	39	CCNCC
5	39	C1CCCNC1
6	38	O=Cc1ccccc1
7	38	CCCCC
8	37	Oc1ccccc1
9	29	CCCCC=O
10	27	OCC(O)C
11	26	O=[S](=O)c1ccccc1
12	25	CCc1ccccc1
13	22	CCCc1ccccc1
14	22	Cc1ccc(cc1)N
15	22	CN1CCNCC1
16	21	CCCC=O
17	21	Nc1ccc(cc1)[S](=O)=O
18	20	CC(CN)O
19	20	CC(N)(C)C
20	20	N[C@@H]1CN2[C@@H]1SC([C@@H]2C)(C)C
21	20	O=C[C@@H]1N2C[C@H]([C@H]2SC1(C)C)N
22	20	O=C[C@@H]1N2C(=O)[C@H]([C@H]2SC1(C)C)N
23	20	O=C1[C@@H](N)[C@@H]2N1[C@@H](C)C(S2)(C)C
24	19	NCC(CO)O
25	18	N[C@@H]1CN2[C@@H]1SC([C@@H]2C(=O)O)(C)C
26	18	OC(=O)[C@@H]1N2C(=O)[C@H]([C@H]2SC1(C)C)N
27	18	OC[C@@H]1N2C(=O)[C@H]([C@H]2SC1(C)C)N
28	18	OC[C@@H]1N2C[C@H]([C@H]2SC1(C)C)N
29	18	CCCN1CCNCC1
30	17	CCCNC
31	17	CCCCc1ccccc1

32	17	c1cccnc1
33	17	CCCCO
34	16	Cc1ccccc1O
35	16	Fc1ccccc1
36	16	CCCCCO
37	16	c1ncc[nH]1
38	16	O=Cc1ccc(cc1)N
39	15	CCN(C)C
40	15	c1ccc(cc1)Cc1ccccc1
41	14	CCCCC(=O)O
42	14	O=CCCCc1ccccc1
43	14	C1CNCCO1
44	14	OCCNCC
45	14	NCc1ccccc1
46	13	Nc1ccccc1
47	13	CCCN(C)C
48	13	O=Cc1ccccc1O
49	13	CNCCO
50	13	OCCN1CCNCC1
51	13	CC=C(C)C
52	13	Clc1ccccc1
53	13	CCc1ccc(cc1)O
54	12	CCC[C@H](N)C
55	12	CC(NCC(O)C)C
56	12	CCC[C@@H](C=O)N
57	12	CCN1CCNCC1
58	12	O=CCc1ccccc1
59	12	CCCCN
60	12	c1ncc2c(n1)[nH]cn2
61	12	Cc1ccc(cc1)O
62	12	OCC(CNC(C)C)O
63	12	CCCCCC
64	11	Oc1ccccc1O
65	11	NCCNCC
66	11	Nc1ncnc2c1nc[nH]2
67	11	C[C@@H](C=O)N
68	11	CCC[C@@H](CO)N
69	11	CCC[C@@H](C(=O)O)N
70	11	OCc1ccccc1
71	11	CCOCC
72	10	C[C@H](CCc1ccccc1)N
73	10	O=CCC[C@@H](C=O)N
74	10	CCCCCN
75	10	O=CCC[C@H](N)C
76	10	N[C@@H]1CN2[C@@H]1SCC(=C2C)C

77	10	Nc1ccccn1
78	10	O=CCC[C@@H](C(=O)O)N
79	10	OCCN(C)C
80	10	CCCc1ccc(cc1)O
81	10	C[C@@H](c1ccccc1)N
82	10	O=CC(C)C
83	10	OCCN(CC)CC
84	10	N[C@H](CO)CCC=O
85	10	CCCC(=O)O
86	10	O=CC1=C(C)CS[C@H]2N1C(=O)[C@H]2N
87	10	O=CC1=C(C)CS[C@H]2N1C[C@H]2N
88	10	O=C1[C@@H](N)[C@@H]2N1C(=C(CS2)C)C
89	10	O=C[C@H](CCc1ccccc1)N
90	10	O=C[C@@H](c1ccccc1)N
91	10	NCC(=O)O
92	10	C1CCCN1
93	10	CCOCc1ccccc1
94	9	Clc1ccc(cc1)Cc1ccccc1
95	9	CO/N=C(/c1cscn1)\C=O
96	9	O=CCCC=O
97	9	Nc1ncccn1
98	9	OCC1=C(C)CS[C@H]2N1C(=O)[C@H]2N
99	9	N[C@@H]1CN2[C@@H]1SCC(=C2C(=O)O)C
100	9	N[C@@H]1CN2[C@@H]1SCC(=C2C=O)CO

Table S9. Top 100 fragments from REACP, k=8, x

Rank	Num	SMILES
1	57	*N1CCN(CC1)*
2	40	*c1ccccc1
3	32	CCN(CC)*
4	22	*OCC(C*)O
5	21	*c1ccc(cc1)S(=O)(=O)*
6	21	Nc1ccc(cc1)S(=O)(=O)*
7	20	*N[C@@H]1C(=*)N2[C@@H]1SC([C@@H]2C(=*)*)(C)C
8	20	*N[C@@H]1C(=O)N2[C@@H]1SC([C@@H]2C(=O)*)(C)C
9	20	*N[C@@H]1C(=O)N2[C@@H]1SC([C@@H]2C(=*)*)(C)C
10	20	*N[C@@H]1C(=*)N2[C@@H]1SC([C@@H]2C(=O)*)(C)C
11	20	*NC(C)(C)C
12	19	*c1ccccc1*
13	19	*N1CCN(CC1)C
14	19	*N1CCCCC1
15	18	*NCC(CO*)O
16	18	*N[C@@H]1C(=O)N2[C@@H]1SC([C@@H]2C(=O)O)(C)C
17	18	*N[C@@H]1C(=*)N2[C@@H]1SC([C@@H]2C(=*)O)(C)C

18	18	*N[C@@H]1C(=O)N2[C@@H]1SC([C@@H]2C(=*)O)(C)C
19	18	*NCC(C*)O
20	18	*N[C@@H]1C(=*)N2[C@@H]1SC([C@@H]2C(=O)O)(C)C
21	16	*n1cncc1
22	14	*c1ccc(cc1)C(=O)*
23	14	*c1ccc(cc1)C(=*)*
24	14	*=CCCCC(=O)*
25	14	*=CCCCC(=*)*
26	14	*CCCN1CCN(CC1)*
27	13	*Cc1ccc(cc1)*
28	13	*N1CCOCC1
29	13	*CCN(C)C
30	13	*OCCN(C)*
31	12	*Cc1ccccc1
32	12	*CC(CNC(C)C)O
33	12	*c1ccc(cc1)F
34	12	*OCC(CNC(C)C)O
35	11	*Oc1ccccc1
36	10	*N[C@H](C(=O)*)CCc1ccccc1
37	10	*N[C@H](C(=*)*)CCc1ccccc1
38	10	*[C@@H](C(=*)*)CCc1ccccc1
39	10	*[C@@H](C(=O)*)CCc1ccccc1
40	10	*OCCN(C)C
41	9	*c1ccc(cc1)Cl
42	9	*Oc1ccccc1O*
43	9	*Oc1ccccc1*
44	9	*Nc1ccc(cc1)C(=O)*
45	9	*Nc1ccc(cc1)C(=*)*
46	9	*OCC1=C(C(=*)*)N2[C@H](SC1)[C@@H](C2=*)N*
47	9	*N[C@H](C(=O)*)C
48	9	*OCC1=C(C(=O)*)N2[C@H](SC1)[C@@H](C2=*)N*
49	9	*OCC1=C(C(=*)*)N2[C@H](SC1)[C@@H](C2=O)N*
50	9	*C(=*)c1ccccc1*
51	9	*C(=*)/C(=N\OC)/c1csc(n1)*
52	9	*C(=O)/C(=N\OC)/c1csc(n1)N
53	9	*C(=O)c1ccccc1*
54	9	*=C/C=C/C(=*)C
55	9	*OCC1=C(C(=O)*)N2[C@H](SC1)[C@@H](C2=O)N*
56	9	*C(=O)/C(=N\OC)/c1csc(n1)*
57	9	*C(=*)/C(=N\OC)/c1csc(n1)N
58	8	*N1CCCC1
59	8	*Nc1c(C)cccc1C
60	8	*Cc1ccc(cc1)c1ccccc1*
61	8	CCOC(=O)[C@H](CCc1ccccc1)N*
62	8	*c1ccccn1

63	8	CCOC(=*)[C@@H](CCc1ccccc1)*
64	8	*c1n[nH]nn1
65	8	CCOC(=*)[C@H](CCc1ccccc1)N*
66	8	CCOC(=O)[C@@H](CCc1ccccc1)*
67	8	CCN(CCO*)CC
68	8	*N(CC)CCO*
69	8	*N[C@H](C(=*)O)CCC(=O)O
70	8	*N[C@H](C(=O)O)CCC(=O)*
71	8	*N[C@H](C(=O)O)CCC(=*)O
72	8	*N[C@H](C(=O)O)CCC(=*)*
73	8	*N[C@H](C(=O)*)CCC(=O)O
74	8	*N[C@H](C(=O)*)CCC(=O)*
75	8	*c1ncnc2c1ncn2*
76	8	Nc1ncnc2c1ncn2*
77	8	*N[C@H](C(=O)*)CCC(=*)O
78	8	*N[C@H](C(=O)*)CCC(=*)*
79	8	*=CC1=C(C)CCCC1(C)C
80	8	*N[C@H](C(=*)O)CCC(=O)*
81	8	*N[C@H](C(=*)O)CCC(=*)O
82	8	*N[C@H](C(=*)O)CCC(=*)*
83	8	*N[C@H](C(=*)*)CCC(=O)O
84	8	*N[C@H](C(=*)*)CCC(=O)*
85	8	*N[C@H](C(=*)*)CCC(=*)O
86	8	*N[C@H](C(=*)*)CCC(=*)*
87	8	*N(Cc1ccccc1)*
88	8	*N[C@H](C(=O)O)CCC(=O)O
89	8	*=C/C(=C/C=C/C(=*)C)/C
90	8	*=C/C=C(/C=*)\C
91	8	*C(=*)c1ccccc1
92	7	*OCC1=C(C(=O)O)N2[C@H](SC1)[C@@H](C2=*)N*
93	7	*n1cncn1
94	7	*C(=*)C(C)(C)C
95	7	*C(=O)C(C)(C)C
96	7	N[C@H](c1ccccc1)C(=O)*
97	7	*[C@H](c1ccccc1)C(=O)*
98	7	N[C@H](c1ccccc1)C(=*)*
99	7	*[C@H](c1ccccc1)C(=*)*
100	7	*CCCN(C)C

Table S10. Top 100 fragments from extendedREACP, k=1

Rank	Num	SMILES
1	811	c1ccccc1
2	85	c1cccnc1
3	74	C1CCCNC1

4	62	C1CCCCC1
5	48	N1CCNCC1
6	43	O=C1C=C[C@H]2C(=C1)CC[C@@H]1[C@@H]2CC[C@H]2[C@H]1CCC2
7	39	c1ncc[nH]1
8	36	C1CCCO1
9	35	C1[C@H]2SCC=CN2C1=O
10	31	c1ccc2c(c1)Sc1c(N2)cccc1
11	31	C1CCCOC1
12	30	O=C1CC[C@H]2C(=C1)CC[C@@H]1[C@@H]2CC[C@H]2[C@H]1CCC2
13	30	c1nccs1
14	29	c1ccncn1
15	27	C1CCCN1
16	24	CN1CCNCC1
17	23	c1cccs1
18	23	O=C1C[C@@H]2N1CCS2
19	22	c1ccc2c(c1)[nH]cc2
20	21	c1ccc2c(c1)[nH]cn2
21	20	C1CCCC1
22	20	c1ccc2c(c1)nccc2
23	18	NCC(=O)O
24	17	O=C1CN=Cc2c(N1)cccc2
25	17	c1ccco1
26	17	c1ncc2c(n1)[nH]cn2
27	16	c1ccc2c(c1)cccc2
28	16	C1CNCCO1
29	15	c1nnn[nH]1
30	15	CCCCC=O
31	15	CN1CCCCC1
32	15	CCCCC
33	14	O=C1CC(=O)NC(=O)N1
34	13	C1CNC=N1
35	12	CCC(=O)O
36	12	c1ccno1
37	12	O=c1cc[nH]c2c1cccc2
38	12	C1CCC=CC1
39	11	O=c1cc[nH]c(=O)[nH]1
40	11	CN1CCCC1
41	11	CCCCCC
42	11	c1ccc2c(c1)ncnc2
43	10	C1C=CNC=C1
44	10	CCCCCC(=O)O
45	10	C1C[C@@H]2[C@@H](C1)[C@@H]1CCc3c([C@H]1CC2)cccc3
46	10	c1ccn[nH]1
47	10	CCCCC(=O)O
48	10	c1ncn[nH]1

49	10	O=CC(C)C
50	9	C1CC[C@H]2[C@H](C1)CCC2
51	9	O=c1nccc[nH]1
52	9	OC(=O)CC[C@@H](C(=O)O)N
53	9	CCCCCCC
54	9	O=C1C=C[C@H]2C(=C1)CC[C@@H]1[C@@H]2CC[C@H]2[C@H]1C[C@@H]1[C@H]2OCO1
55	9	n1ncn[nH]1
56	9	O=C1C=CC[C@H]2[C@@H]1C=C1[C@H](C2)Cc2c(C1=O)cccc2
57	9	O=C1NCC(=O)N1
58	8	O=c1[nH]c(=O)c2c([nH]1)nc[nH]2
59	8	C[C@@H](C(=O)O)N
60	8	CC(F)(F)F
61	8	C=C1CCCCC1
62	7	O=C1NCCO1
63	7	CCNCC
64	7	C1CCc2c(C1)cccc2
65	7	CCCC=O
66	7	O=CC(C)(C)C
67	6	CCCC(=O)O
68	6	O=c1nc[nH]c2c1nc[nH]2
69	6	c1ccc2c(c1)Nc1ccccc1CC2
70	6	CN1CCC=C2[C@H]1Cc1c[nH]c3c1c2ccc3
71	6	C1C2CC3CC1CC(C2)C3
72	6	O=c1[nH]cnc2c1nc[nH]2
73	6	O=c1cc[nH]c2c1cccn2
74	6	C1CCCCNC1
75	6	c1ccc[nH+]c1
76	5	CC(C(=O)O)C
77	5	C1CCC[NH2+]C1
78	5	n1ccncc1
79	5	C1Oc2c(O1)cccc2
80	5	CC(N)(C)C
81	5	C1[C@H]2CC=CN2C1=O
82	5	O=CCCC(=O)O
83	5	c1ccc2c(c1)Sc1c(C2)cccc1
84	5	O=C1c2cc3CCCCc3cc2C(=O)c2c1cccc2
85	5	CCCC(C)C
86	5	NCNc1ccccc1
87	5	C(C#N)C=O
88	5	CN1[C@@H]2CCC[C@H]1CC2
89	5	C1CN2CCC1CC2
90	5	N1CCc2c(C1)cccc2
91	5	C1Cc2c(C1)cccc2
92	5	c1ccc[nH]1

93	5	O=C1CCCC(=O)N1
94	4	CN1CC[C@@]23[C@@H]4[C@H]1Cc1c3c(O[C@H]2CC=C4)ccc1
95	4	CN1CC[C@@]23[C@@H]4[C@H]1Cc1c3c(O[C@H]2C(=O)CC4)ccc1
96	4	C1CCc2c(O1)cccc2
97	4	C1C[C@@H]2CC[C@H](C1)[NH2+]2
98	4	c1ccc2c(c1)Cc1c2cccc1
99	4	C1CCC[NH2+]1
100	4	O=c1ccoc2c1cccc2

Table S11. Top 100 fragments from extendedREACP, k=8

Rank	Num	SMILES
1	811	c1ccccc1
2	483	Cc1ccccc1
3	209	Oc1ccccc1
4	156	Nc1ccccc1
5	131	O=Cc1ccccc1
6	129	Clc1ccccc1
7	102	OCc1ccccc1
8	101	CCc1ccccc1
9	85	c1cccnc1
10	79	Cc1ccc(cc1)O
11	74	C1CCCNC1
12	73	COc1ccccc1
13	69	Fc1ccccc1
14	62	C1CCCCC1
15	62	O=[S](=O)c1ccccc1
16	61	c1ccc(cc1)Cc1ccccc1
17	55	CCCc1ccccc1
18	54	CCNCC
19	52	NS(=O)(=O)c1ccccc1
20	52	CCC(=O)O
21	51	O=CCc1ccccc1
22	48	N1CCNCC1
23	44	Cc1cccc(c1)O
24	43	O=C[C@H]1CC[C@@H]2[C@@H]1C[C@H](O)[C@H]1[C@H]2CCC2=CC(=O)C=C[C@H]12
25	43	NCc1ccccc1
26	43	O=C1C=C[C@H]2C(=C1)CC[C@@H]1[C@@H]2CC[C@H]2[C@H]1CCC2
27	43	O=C1C=C[C@H]2C(=C1)CC[C@@H]1[C@@H]2[C@@H](O)C[C@]2([C@H]1CCC2)C
28	43	O=C1C=C[C@H]2C(=C1)CC[C@@H]1[C@@H]2CC[C@]2([C@H]1CCC2)C
29	43	O=C1C=C[C@H]2C(=C1)CC[C@@H]1[C@@H]2[C@@H](O)C[C@H]2[C@H]1CCC2
30	43	O=C[C@H]1CC[C@@H]2[C@@H]1CC[C@H]1[C@H]2CCC2=CC(=O)C=C[C@H]12
31	43	O=C1C=C[C@]2(C(=C1)CC[C@@H]1[C@@H]2CC[C@H]2[C@H]1CCC2)C

32	43	O=C[C@H]1CC[C@@H]2[C@@H]1CC[C@H]1[C@H]2CCC2=CC(=O)C=C[C@]12C
33	43	O=C[C@H]1CC[C@@H]2[C@@H]1C[C@H](O)[C@H]1[C@H]2CCC2=CC(=O)C=C[C@]12C
34	43	O=C[C@H]1CC[C@@H]2[C@]1(C)CC[C@H]1[C@H]2CCC2=CC(=O)C=C[C@H]12
35	43	O=C[C@H]1CC[C@@H]2[C@]1(C)CC[C@H]1[C@H]2CCC2=CC(=O)C=C[C@]12C
36	43	O=C1C=C[C@]2(C(=C1)CC[C@@H]1[C@@H]2CC[C@]2([C@H]1CCC2)C)C
37	43	O=C[C@H]1CC[C@@H]2[C@]1(C)C[C@H](O)[C@H]1[C@H]2CCC2=CC(=O)C=C[C@]12C
38	43	O=C1C=C[C@]2(C(=C1)CC[C@@H]1[C@@H]2[C@@H](O)C[C@H]2[C@H]1CCC2)C
39	43	O=C[C@H]1CC[C@@H]2[C@]1(C)C[C@H](O)[C@H]1[C@H]2CCC2=CC(=O)C=C[C@H]12
40	43	O=C1C=C[C@]2(C(=C1)CC[C@@H]1[C@@H]2[C@@H](O)C[C@]2([C@H]1CCC2)C)C
41	39	Cc1cccc(c1)C
42	39	CCOC=O
43	39	c1ncc[nH]1
44	38	O=C1C=C[C@]2(C(=C1)CC[C@@H]1[C@@H]2CC[C@]2([C@H]1CC[C@H]2O)C)C
45	38	O=C1C=C[C@H]2C(=C1)CC[C@@H]1[C@@H]2[C@@H](O)C[C@H]2[C@H]1CC[C@H]2O
46	38	O=C1C=C[C@H]2C(=C1)CC[C@@H]1[C@@H]2CC[C@]2([C@H]1CC[C@H]2O)C
47	38	O=C1C=C[C@H]2C(=C1)CC[C@@H]1[C@@H]2CC[C@H]2[C@H]1CC[C@H]2O
48	38	O=C1C=C[C@H]2C(=C1)CC[C@@H]1[C@@H]2[C@@H](O)C[C@]2([C@H]1CC[C@H]2O)C
49	38	O=C1C=C[C@]2(C(=C1)CC[C@@H]1[C@@H]2CC[C@H]2[C@H]1CC[C@H]2O)C
50	38	O=C[C@@]1(O)CC[C@@H]2[C@@H]1CC[C@H]1[C@H]2CCC2=CC(=O)C=C[C@]12C
51	38	O=C1C=C[C@]2(C(=C1)CC[C@@H]1[C@@H]2[C@@H](O)C[C@H]2[C@H]1CC[C@H]2O)C
52	38	O=C1C=C[C@]2(C(=C1)CC[C@@H]1[C@@H]2[C@@H](O)C[C@]2([C@H]1CC[C@H]2O)C)C
53	38	O=C[C@@]1(O)CC[C@@H]2[C@@H]1CC[C@H]1[C@H]2CCC2=CC(=O)C=C[C@H]12
54	38	O=C[C@@]1(O)CC[C@@H]2[C@]1(C)C[C@H](O)[C@H]1[C@H]2CCC2=CC(=O)C=C[C@]12C
55	38	O=C[C@@]1(O)CC[C@@H]2[C@]1(C)C[C@H](O)[C@H]1[C@H]2CCC2=CC(=O)C=C[C@H]12
56	38	O=C[C@@]1(O)CC[C@@H]2[C@]1(C)CC[C@H]1[C@H]2CCC2=CC(=O)C=C[C@]12C
57	38	O=C[C@@]1(O)CC[C@@H]2[C@]1(C)CC[C@H]1[C@H]2CCC2=CC(=O)C=C[C@H]12
58	38	O=C[C@@]1(O)CC[C@@H]2[C@@H]1C[C@H](O)[C@H]1[C@H]2CCC2=CC(=O)C=C[C@]12C
59	38	O=C[C@@]1(O)CC[C@@H]2[C@@H]1C[C@H](O)[C@H]1[C@H]2CCC2=CC(=O)C=C[C@H]12
60	38	CCN(C)C
61	36	Cc1ccccn1
62	36	CN1CCNCC1
63	36	C1CCCO1

64	35	C1[C@H]2SCC=CN2C1=O
65	35	O=CC1=CCS[C@H]2N1C(=O)C2
66	35	CCCOc1ccccc1
67	34	N[C@H]1[C@H]2SCC=CN2C1=O
68	34	O=CC1=CCS[C@H]2N1C(=O)[C@H]2N
69	34	OCC(O)C
70	33	NCCc1ccccc1
71	32	OCC(=O)[C@H]1CC[C@@H]2[C@]1(C)C[C@H](O)[C@H]1[C@H]2CCC2=CC(=O)C=C[C@]12C
72	32	Cc1ccc(cc1)Cl
73	32	OCC(=O)[C@H]1CC[C@@H]2[C@@H]1C[C@H](O)[C@H]1[C@H]2CCC2=CC(=O)C=C[C@]12C
74	32	OCC(=O)[C@H]1CC[C@@H]2[C@]1(C)CC[C@H]1[C@H]2CCC2=CC(=O)C=C[C@H]12
75	32	OCC(=O)[C@H]1CC[C@@H]2[C@@H]1C[C@H](O)[C@H]1[C@H]2CCC2=CC(=O)C=C[C@H]12
76	32	OCC(=O)[C@H]1CC[C@@H]2[C@]1(C)CC[C@H]1[C@H]2CCC2=CC(=O)C=C[C@]12C
77	32	OCC(=O)[C@H]1CC[C@@H]2[C@]1(C)C[C@H](O)[C@H]1[C@H]2CCC2=CC(=O)C=C[C@H]12
78	32	OCC(=O)[C@H]1CC[C@@H]2[C@@H]1CC[C@H]1[C@H]2CCC2=CC(=O)C=C[C@H]12
79	32	Clc1cccc(c1)Cl
80	32	OCC(=O)[C@H]1CC[C@@H]2[C@@H]1CC[C@H]1[C@H]2CCC2=CC(=O)C=C[C@]12C
81	31	OC(=O)c1ccccc1
82	31	C1CCCOC1
83	31	c1ccc2c(c1)Sc1c(N2)cccc1
84	31	NC(=O)Cc1ccccc1
85	30	O=C1[C@@H](N)[C@@H]2N1C(=CCS2)C(=O)O
86	30	O=C1CC[C@H]2C(=C1)CC[C@@H]1[C@@H]2CC[C@]2([C@H]1CCC2)C
87	30	Oc1ccccc1O
88	30	O=C1CC[C@H]2C(=C1)CC[C@@H]1[C@@H]2CC[C@H]2[C@H]1CCC2
89	30	CC1=CN2[C@H](SC1)CC2=O
90	30	c1nccs1
91	30	O=CN[C@@H]1C(=O)N2[C@@H]1SCC=C2C=O
92	30	O=CN[C@@H]1C(=O)N2[C@@H]1SCC=C2
93	30	O=C1C[C@@H]2N1C(=CCS2)C(=O)O
94	30	O=CC1=C(C)CS[C@H]2N1C(=O)C2
95	29	OC(=O)Cc1ccccc1
96	29	O=CC1=C(C)CS[C@H]2N1C(=O)[C@H]2N
97	29	c1ccncn1
98	29	CC1=CN2[C@H](SC1)[C@@H](C2=O)N
99	28	OCC(=O)[C@@]1(O)CC[C@@H]2[C@@H]1C[C@H](O)[C@H]1[C@H]2CCC2=CC(=O)C=C[C@H]12
100	28	O=CNc1ccccc1

Table S12. Top 100 fragments from extendedREACP, k=8, x

Rank	Num	SMILES
1	296	*c1ccccc1
2	295	*c1ccc(cc1)*
3	133	*c1ccc(c(c1)*)*
4	91	*c1ccccc1*
5	76	*Cc1ccccc1
6	74	*Cc1ccc(cc1)*
7	70	*C(c1ccccc1)*
8	51	*Oc1ccc(cc1)*
9	51	*c1ccc(cc1)Cl
10	49	*c1ccc(cc1)C(=O)*
11	48	*c1ccc(cc1)C(*)*
12	46	*c1cccc(c1)*
13	43	*c1ccc(cc1)S(=O)(=O)*
14	41	*c1c(*)cccc1*
15	39	*N1CCN(CC1)*
16	34	*c1ccc(c(c1)*)O
17	33	*C(c1ccccc1)(*)*
18	33	CCN(CC)*
19	32	*c1ccc(cc1)F
20	30	*c1ccc(cc1)N
21	30	*NS(=O)(=O)c1ccc(cc1)*
22	29	*Nc1ccc(cc1)*
23	29	*c1ccc(cc1)O
24	29	*N[C@@H]1C(=O)N2[C@@H]1SCC(=C2*)*
25	29	*N[C@@H]1C(=O)N2[C@@H]1SCC(=C2C(=O)*)*
26	28	*CCN(C)C
27	28	*c1ccccn1
28	26	*C(=O)N[C@@H]1C(=O)N2[C@@H]1SCC(=C2*)*
29	26	*C1CCCCC1
30	26	*C(=O)N[C@@H]1C(=O)N2[C@@H]1SCC(=C2C(=O)*)*
31	26	*OCC(C*)O
32	25	*N[C@@H]1C(=O)N2[C@@H]1SCC(=C2C(=O)O)*
33	25	*c1ccc2c(c1)N(*)c1c(S2)cccc1
34	24	Nc1ccc(cc1)S(=O)(=O)*
35	24	*c1ccc(c(c1)O)*
36	24	*N1CCN(CC1)C
37	23	O=C1[C@H](*)[C@@H]2N1C(=C(CS2)*)*
38	23	O=C1[C@H](*)[C@@H]2N1C(=C(CS2)*)C(=O)*
39	23	*c1ccc(c(c1)Cl)*
40	23	*c1scc(n1)*
41	22	*C(=O)c1ccccc1*
42	22	*C(=O)N[C@@H]1C(=O)N2[C@@H]1SCC(=C2C(=O)O)*
43	22	*C(=O)OCC

44	21	*C(c1ccc(c(c1)*)*)*
45	21	*c1cc(*)c(c(c1)*)*
46	20	*N[C@@H]1C(=O)N2[C@@H]1SC([C@@H]2C(=O)*)(*)C
47	20	*N1CCCCC1
48	20	*N[C@@H]1C(=O)N2[C@@H]1SCC(=C2*)C*
49	20	*N[C@@H]1C(=O)N2[C@@H]1SC([C@@H]2C(=O)*)(C)C
50	20	*N[C@@H]1C(=O)N2[C@@H]1SCC(=C2C(=O)*)C*
51	20	*N[C@@H]1C(=O)N2[C@@H]1SC([C@@H]2C(=O)*)(*)*
52	20	*NC(C)(C)C
53	19	*N[C@@H]1C(=O)N2[C@@H]1SC([C@@H]2*)(*)*
54	19	*CC1=C(*)N2[C@H](SC1)[C@@H](C2=O)*
55	19	*c1c(*)cccc1Cl
56	19	*C(=O)N[C@@H]1C(=O)N2[C@@H]1SC([C@@H]2C(=O)*)(*)C
57	19	*NCC(CO*)*
58	19	*C(=O)N[C@@H]1C(=O)N2[C@@H]1SC([C@@H]2C(=O)*)(C)C
59	19	O=C1[C@H](*)[C@@H]2N1C(=C(CS2)*)C(=O)O
60	19	*N[C@@H]1C(=O)N2[C@@H]1SC([C@@H]2*)(*)C
61	19	*CC1=C(C(=O)*)N2[C@H](SC1)[C@@H](C2=O)*
62	19	*NCC(C*)O
63	19	*NCC(CO*)O
64	19	*N[C@@H]1C(=O)N2[C@@H]1SC([C@@H]2*)(C)C
65	19	*C(=O)N[C@@H]1C(=O)N2[C@@H]1SC([C@@H]2C(=O)*)(*)*
66	18	*C1CCN(CC1)*
67	18	*N[C@@H]1C(=O)N2[C@@H]1SC([C@@H]2C(=O)O)(*)C
68	18	*C(=O)N[C@@H]1C(=O)N2[C@@H]1SC([C@@H]2*)(C)C
69	18	*c1ccc(c(c1)O)O
70	18	*C(=O)N[C@@H]1C(=O)N2[C@@H]1SC([C@@H]2*)(*)*
71	18	*N[C@@H]1C(=O)N2[C@@H]1SC([C@@H]2C(=O)O)(C)C
72	18	*N[C@@H]1C(=O)N2[C@@H]1SC([C@@H]2C(=O)O)(*)*
73	18	*C(=O)N[C@@H]1C(=O)N2[C@@H]1SC([C@@H]2*)(*)C
74	18	*C(c1ccccc1)(O)*
75	18	*C(=O)c1ccccc1
76	17	*CCOC(=O)*
77	17	*COC(=O)C
78	17	*OP(=O)(O)O
79	17	Clc1ccc(c(c1)*)*
80	17	*NS(=O)(=O)c1ccc(cc1)N
81	17	*CC1=C(C(=O)*)N2[C@H](SC1)[C@@H](C2=O)NC(=O)*
82	17	*C(c1ccc(c(c1)*)O)*
83	17	*Cc1ccc(c(c1)*)*
84	17	*C(c1ccccc1)(c1ccccc1)*
85	17	*Nc1ccc(c(c1)*)*
86	17	*C(=O)N[C@@H]1C(=O)N2[C@@H]1SC([C@@H]2C(=O)O)(*)C
87	17	*CC1=C(*)N2[C@H](SC1)[C@@H](C2=O)NC(=O)*
88	17	*C(=O)N[C@@H]1C(=O)N2[C@@H]1SC([C@@H]2C(=O)O)(*)*

89	17	*C(=O)N[C@@H]1C(=O)N2[C@@H]1SC([C@@H]2C(=O)O)(C)C
90	16	*c1ccc(c(c1)F)*
91	16	*Oc1ccc(cc1*)*
92	16	*CCCN1c2ccccc2Sc2c1cc(*)cc2
93	16	*N[C@@H]1C(=O)N2[C@@H]1SCC(=C2C(=O)O)C*
94	16	*c1csc(n1)N
95	16	*Oc1ccccc1*
96	16	*c1ccc(cc1)C(=*)*
97	16	*c1ccccc1Cl
98	16	*Cc1ccc(cc1)O*
99	16	*n1cncc1
100	16	*[C@H](c1ccccc1)C(=O)*

Tables S1-S12. Top ranked fragments. The number of occurrences (Num) and the SMILES of the 100 most frequent fragments are reported. Data are shown for simple fragmentation (k=1) and exhaustive fragmentation with (k=8, x) and without dummy atoms (k=8) for the four fragmentation methods BRICS (**S1-S3**), CCQ (**S4-S6**), RECAP (**S7-S9**) and extendedRECAP (**S10-S12**).
